# Supplementary material for: Sustainability of a school-based health intervention for prevention of non-communicable diseases in marginalised communities: protocol for a mixed-methods cohort study
Source: BMJ Open. 2021 Oct 4;11(10):e047296. doi: 10.1136/bmjopen-2020-047296 (PMC8493924; doi:10.1136/bmjopen-2020-047296)
Supplement: Supplementary data [file bmjopen-2020-047296supp001.pdf]

Sustainability of a school-based health intervention for prevention of non-communicable diseases in marginalised communities: protocol for a mixed methods cohort study

Arnaiz, Adams et al.

Supplementary Online Material

Information on COVID-19-Related School Lockdown and Closure in South Africa and the Port Elizabeth Region

On 15 March 2020, the President of South Africa, Cyril Ramaphosa, declared a national state of disaster in response to the COVID-19 global pandemic. Ramaphosa announced measures such as immediate travel restrictions and the closure of all schools from 18 March. On 23 March, a national lockdown was announced lasting for 21 days, starting on 27 March. The initial three-week period was eventually extended, with different levels of lockdown regulating the various kinds of restrictions placed people's movement and activity. South Africa developed a 5-tier "Risk Adjusted Strategy Lockdown" categorization [i.e., level 1 (least severe) to level 5 (total lockdown)]. On 01 May 2020, Ramaphosa announced that a gradual and phased easing of lockdown restrictions would be implemented, lowering the national alert level to 4. From 01 June, the national restrictions were lowered to level 3. The restrictions were further lowered to alert level 2 on 17 August. From 21 September, restrictions were lowered again to alert level 1. In December, the country experienced a second wave of COVID-19 infections, resulting in a lockdown level tightening from an adjusted level 1 to an adjusted level 3, starting on 29 December. However, on 01 March 2021, the lockdown level was lowered again from an adjusted level 3 to an adjusted level 1. On 31 May 2021 the country was moved from adjusted level 1 to an adjusted alert level 2, due to a third wave of infections. On 15 June 2021 the country was moved to alert level 3. On 28 June 2021, the country was moved to adjusted level 4, with the Delta variant fast becoming the dominant strain in the country. On 25 July the country was moved to adjusted level 3.

The COVID-19 pandemic has affected education systems globally, leading to the near complete closure of some schools. South African schools were closed on 18 March 2020, resuming early June 2020. To compensate for the loss in educational teaching time, the mid-year school holidays were shortened by one week. However, for 10 weeks, children were not permitted to go to school. Although a small proportion of schools could quickly adapt and switch to online learning, educational activity came to a complete stop for most schools and no learning and teaching could take place, especially for quintile three categorization schools and below (i.e., no fee-paying schools), such as those involved in this study. Accordingly, the deep inequalities within the South African schooling system became evident. After several delays, schools officially reopened on 08 June 2020, with a planned staggered return of grades. Learners who returned to school first were those at exit points of primary and high schools, i.e., grades 7 and 12, respectively. However, on 24 July 2020, with only

five grades having returned (three for less than one full month), schools were closed again due to an upsurge in COVID-19 cases and deaths. Although schools remained closed until 24 August 2020, grade 12 learners returned to schools on 03 August 2020. On 11 August 2020, grade 7 learners returned to school, whereas on 24 August 2020, grades R – 4, grade 6, and grades 9 – 11 returned to school. Lastly, grades 5 and 8 returned to school on 31 August 2020.

The most significant educational time loss came from the timetabling model that schools were forced to adopt to meet the social distancing requirements, in addition with the staggered return to school system to further limit the spread of COVID-19. The Department of Basic Education stated that each school must comply with the 1.5m social distancing requirements, as well as all schools must operate at 50%, or less, of their capacity at any given time. Schools were given the liberty to decide how this would be implemented and many schools had varied approaches, incorporating what the schools referred to as a block teaching system. The following were some examples of how the implementation/grouping of the 50% capacity occurred:

- Each class was split in half and would alternate the days learners attend school.
- The entire grade was split in half and would alternate the days each class would attend school.
- Junior and senior grades were split, whereby the junior and senior grades would alternate the days attending school, i.e., all grades 4 and 5 on one day and all grades 6 and 7 the next day.
- A ratio of 3:2 days out of 5 school days was incorporated, whereby one group of learners attended school for the first three days of the week, and then the last 2 days of the following week, and vice versa for the second group.

This 50% capacity system is currently still applicable and being implemented at all schools, particularly those schools involved in this study. Schools were also advised that they should not exclude any of the subjects from the curriculum, but that they should adapt the timetable how they deem it necessary by prioritizing the more heavily weighted academic subjects, such as English, Afrikaans, Mathematics and Science. Consequently, the allocation of certain subjects would have been affected. Furthermore, given that physical education is currently not a stand-alone subject and forms part of the Life Orientation/Life Skills subject, the likelihood of physical education receiving limited attention is high. Although some schools may have attempted to include the Life Orientation/Life Skills subject in the timetable, our assumption is that it may not have been prioritized and may have been neglected by many schools throughout the COVID-19 pandemic. Hence, we believe that the implementation of the *KaziKidz* material would also not necessarily have taken place as originally planned; and requires further exploration.

Although the initial objective of the study was to examine the long-term effectiveness and sustainability of both the *KaziKidz* and *KaziHealth* programs in promoting long lasting, positive lifestyle changes among children and teachers, the objective has now been adapted to evaluate sustainability concerning the continuation of the program

implementation, without the assistance from the research team, amidst the COVID-19 pandemic. Therefore, the intervention itself has not been adapted, as the study aims to evaluate its sustainability, whilst embracing the COVID-19 reality.

Lockdown level	Description	Duration
**Level 5**	**Total lockdown**: Drastic measures to contain spread of COVID-19 virus and save lives	26 March 2020 – 30 April 2020
**Level 4**	Extreme precautions to limit community transmission and outbreaks, while allowing some activity to resume	01 May 2020 – 31 May 2020
**Level 3**	Restrictions on many activities, including at workplaces and socially, to address a high risk of transmission	01 June 2020 – 17 August 2020
**Level 2**	Physical distancing and restrictions on leisure and social activities to prevent the resurgence of the virus	18 August 2020 – 20 September 2020
**Level 1**	Most normal activity can resume, with precautions and guidelines followed at all times. Population prepared for an increase in alert levels, if necessary	21 September 2020 – 28 December 2020
**Level 3**	Restrictions on many activities, including at workplaces and socially, to address a high risk of transmission	29 December 2020 – 28 February 2021
**Level 1**	Most normal activity can resume, with precautions and guidelines followed at all times. Population prepared for an increase in alert levels, if necessary	01 March 2021 – 30 May 2021
**Level 2**	Physical distancing and restrictions on leisure and social activities to prevent the resurgence of the virus	31 May 2021 – 15 June 2021
**Level 3**	Restrictions on many activities, including at workplaces and socially, to address a high risk of transmission	16 June 2021 – 27 June 2021
**Level 4**	Extreme precautions to limit community transmission and outbreaks, while allowing some activity to resume	28 June 2021 – 24 July 2021
**Level 3**	Restrictions on many activities, including at workplaces and socially, to address a high risk of transmission	25 July 2021 – Present (August 2021)

**2020 Amended School Calendar**		
**Term**	**Duration**	**Number of school days**

Arnaiz P, *et al. BMJ Open* 2022; 11:e047296. doi: 10.1136/bmjopen-2020-047296

1	**15 January – 18 March 2020**	**46**
2	**08 June – 24 July 2020**	**34**
3	**24 August – 23 October 2020**	**44**
	• 03 – 07 August Grade 12 learners returned	
	• 11 – 14 August: Grade 7 learners returned	
	• 17 – 21 August: Grades 7 and 12 learners returned	
	• 24 August: Grades R – 4, Grade 6 and Grades 9 – 11 learners returned	
	• 31 August: Grades 5 and 8 learners returned	
4	**02 November – 15 December 2020**	**32**

2021 Amended School Calendar		
**Term**	**Duration**	**Number of school days**
1	**15 February – 23 April 2021**	**47**
2	**03 May – 09 July 2021**	**39**
3	**26 July – 01 October 2021**	**48**
4	**11 October –15 December 2021**	**48**

Arnaiz P, *et al. BMJ Open* 2022; 11:e047296. doi: 10.1136/bmjopen-2020-047296
