# Supplementary material for: Sustainability of a school-based health intervention for prevention of non-communicable diseases in marginalised communities: protocol for a mixed-methods cohort study
Source: BMJ Open. 2021 Oct 4;11(10):e047296. doi: 10.1136/bmjopen-2020-047296 (PMC8493924; doi:10.1136/bmjopen-2020-047296)
Supplement: Author's manuscript [file bmjopen-2020-047296.draft_revisions.pdf]

BMJ Open is committed to open peer review. As part of this commitment we make the peer review history of every article we publish publicly available.

When an article is published we post the peer reviewers' comments and the authors' responses online. We also post the versions of the paper that were used during peer review. These are the versions that the peer review comments apply to.

The versions of the paper that follow are the versions that were submitted during the peer review process. They are not the versions of record or the final published versions. They should not be cited or distributed as the published version of this manuscript.

BMJ Open is an open access journal and the full, final, typeset and author-corrected version of record of the manuscript is available on our site with no access controls, subscription charges or pay-per-view fees (http://bmjopen.bmj.com).

If you have any questions on BMJ Open's open peer review process please email info.bmjopen@bmj.com

BMJ Open

Sustainability of a health promotion intervention in marginalized schools with children and teachers at risk for non-communicable diseases: protocol for a mixed methods cohort study

Journal:	*BMJ Open*
Manuscript ID	bmjopen-2020-047296
Article Type:	Original research
Date Submitted by the Author:	24-Nov-2020
Complete List of Authors:	Arnaiz, Patricia; University of Basel, Department of Sport, Exercise and Health<br>Adams, Larissa; Nelson Mandela Metropolitan University, Department of Human Movement Science<br>Müller, Ivan; University of Basel, Department of Sport, Exercise and Health<br>Gerber, Markus; University of Basel, Department of Sport, Exercise and Health<br>Walter, Cheryl; Nelson Mandela Metropolitan University, Department of Human Movement Science<br>du Randt, Rosa; Nelson Mandela Metropolitan University, Department of Human Movement Science<br>Steinmann, Peter ; Swiss Tropical and Public Health Institute; University of Basel<br>Bergman, Manfred; University of Basel, Department of Social Sciences<br>Seelig, Harald; University of Basel, Department of Sport, Exercise and Health<br>van Greunen, Darelle; Nelson Mandela Metropolitan University, Centre for Community Technologies<br>Utzinger, Jürg; Swiss Tropical and Public Health Institute; University of Basel<br>Pühse, Uwe ; University of Basel, Department of Sport, Exercise and Health
Keywords:	PREVENTIVE MEDICINE, Community child health < PAEDIATRICS, SPORTS MEDICINE, PUBLIC HEALTH

SCHOLARONE™
Manuscripts

I, the Submitting Author has the right to grant and does grant on behalf of all authors of the Work (as defined in the below author licence), an exclusive licence and/or a non-exclusive licence for contributions from authors who are: i) UK Crown employees; ii) where BMJ has agreed a CC-BY licence shall apply, and/or iii) in accordance with the terms applicable for US Federal Government officers or employees acting as part of their official duties; on a worldwide, perpetual, irrevocable, royalty-free basis to BMJ Publishing Group Ltd ("BMJ") its licensees and where the relevant Journal is co-owned by BMJ to the co-owners of the Journal, to publish the Work in this journal and any other BMJ products and to exploit all rights, as set out in our licence.

The Submitting Author accepts and understands that any supply made under these terms is made by BMJ to the Submitting Author unless you are acting as an employee on behalf of your employer or a postgraduate student of an affiliated institution which is paying any applicable article publishing charge ("APC") for Open Access articles. Where the Submitting Author wishes to make the Work available on an Open Access basis (and intends to pay the relevant APC), the terms of reuse of such Open Access shall be governed by a Creative Commons licence – details of these licences and which Creative Commons licence will apply to this Work are set out in our licence referred to above.

Other than as permitted in any relevant BMJ Author's Self Archiving Policies, I confirm this Work has not been accepted for publication elsewhere, is not being considered for publication elsewhere and does not duplicate material already published. I confirm all authors consent to publication of this Work and authorise the granting of this licence.

For peer review only

Sustainability of a health promotion intervention in marginalized schools with children and teachers at risk for non-communicable diseases: protocol for a mixed methods cohort study

Patricia Arnaiz[1] (PhD candidate, patricia.arnaiz@unibas.ch, https://orcid.org/0000-0001-5626-3510), Larissa Adams[2] (PhD candidate, larissa.adams@mandela.ac.za, https://orcid.org/0000-0003-1459-1916), Ivan Müller[1] (Research associate, ivan.mueller@unibas.ch, https://orcid.org/0000-0002-6397-9979), Markus Gerber[1] (Associate professor, markus.gerber@unibas.ch, https://orcid.org/0000-0001-6140-8948), Cheryl Walter[2] (Head of Department, cheryl.walter@mandela.ac.za), Rosa du Randt[2] (Emeritus professor, rosa.durandt@mandela.ac.za), Peter Steinmann[3,4] (Project leader, peter.steinmann@swisstph.ch, https://orcid.org/0000-0003-4800-3019), Manfred Max Bergman[5] (Professor, max.bergman@unibas.ch, https://orcid.org/0000-0002-4059-5833), Harald Seelig[1] (Statistician, harald.seelig@unibas.ch, https://orcid.org/0000-0002-2178-4807), Darelle van Greunen[6] (Director of Centre, darelle.vangreunen@mandela.ac.za, https://orcid.org/0000-0002-0761-713X), Jürg Utzinger[3,4] (Director of Institute, juerg.utzinger@swisstph.ch), Uwe Pühse[1] (Head of Department, uwe.puehse@unibas.ch, https://orcid.org/0000-0003-1228-316X)

[1]Department of Sport, Exercise and Health, University of Basel, 4052 Basel, Switzerland.
[2]Department of Human Movement Science, Nelson Mandela University, 6031 Port Elizabeth, South Africa.
[3]Swiss Tropical and Public Health Institute, 4002 Basel, Switzerland.
[4]University of Basel, 4001 Basel, Switzerland.
[5]Department of Social Sciences, University of Basel, 4051 Basel, Switzerland.
[6]Centre for Community Technologies, Nelson Mandela University, 6031 Port Elizabeth, South Africa.

Joint first authorship
PA and LA contributed equally to this paper.

Correspondence to
Patricia Arnaiz patricia.arnaiz@unibas.ch Department of Sport, Exercise and Health, University of Basel, Villa Burckhardt, Gellertstrasse 156, 4052 Basel, Switzerland

Word count 3,599.

Number of tables and figures

For peer review only

Tables 1 and 2, figure 1.

ABSTRACT

Introduction There is a steady increase in the prevalence of chronic, lifestyle related diseases both in adults and children from low-and middle-income countries. However, despite the effectiveness of community-based lifestyle interventions to address this, the benefits thereof are likely to disappear, long-term, due to a lack of maintenance. Furthermore, limited knowledge exists on the feasibility of educational programmes in school settings, especially among disadvantaged and high-risk populations. Based on the experience from the *KaziBantu* project, the present study will evaluate the effectiveness, impact, and sustainability of two school-based health intervention programmes, *KaziKidz* and *KaziHealth*, in promoting long-lasting, positive lifestyle changes among children and teachers in disadvantaged schools in Nelson Mandela Bay, South Africa.

Methods and analysis This study has an observational, two-year longitudinal, mixed methods design. It will be conducted in eight quintile three primary schools participating in *KaziBantu* and will follow-up a cohort of 361 children (aged 8 to 16 years) identified as having an increased risk for non-communicable diseases, as well as the 150 participating teachers. Data collection includes quantitative and qualitative methods, such as anthropometric measurements, questionnaires, interviews, and focus group discussions. Analysis will encompass prevalence of health parameters, descriptive frequencies of self-reported health status, health behaviours and quality of life and the longitudinal association of these, perceptions of programmes adoption and acceptance, and an impact analysis based on the RE-AIM framework.

Discussion In settings where resources are scarce, sustainable and effective implementation of prevention programmes are needed. The purpose of this paper is to outline the design of a study to evaluate the feasibility of *KaziKidz* and *KaziHealth* in terms of effectiveness, addressing school children and their teachers, being long-lasting, and becoming institutionalized. We hypothesize that a comprehensive approach might increase implementation feasibility and lead to sustainable favourable health outcomes, generating evidence for policy.

Trial registration number ISRCTN15648510.

STRENGTHS AND LIMITATIONS OF THIS STUDY

- The long-term design of the study will contribute to the limited evidence available on the effectiveness and acceptance of health-promotion interventions among high-risk populations from disadvantaged communities.
- The mixed methods approach, strengthened by the involvement of the community and key stakeholders, will provide a comprehensive and rigorous evaluation of the programme.
- The cohort of children has been carefully selected due to their high-risk profile and the follow-up of personal behavioural changes and health outcomes does not involve a comparison with their non-risk counterparts.
- The results of the study will be context-specific and will allow for refinement of the implementation schema to increase the interventions' feasibility and

For peer review only

generalizability.

- This study will inform local policy and practice to invest in feasible and sustainable evidence-based prevention programmes that promote healthy lifestyles and enhance overall community well-being.

INTRODUCTION

Socioeconomic, environmental, and cultural factors exert a significant influence on children's health and well-being.[1] In impoverished communities in low-and middle-income countries (LMICs), children are especially vulnerable to illness and lags in age-appropriate development, negatively affecting their life prospects.[2-4] Furthermore, the disease profile of urban populations from LMICs is approaching that of high-income countries, as the proportion of deaths attributable to chronic, lifestyle-related diseases increases.[5-7] Of particular concern are changing dietary habits and sedentary lifestyle patterns,[8,9] both known to lead to metabolic and physiological changes, such as obesity and hypertension, even in young children.[10,11] Children presenting with cardiometabolic risk factors are particularly at high risk because of their predisposition to developing non-communicable diseases (NCDs) later in life.[12,13] Thus, a reduction of undesired behaviours could potentially prevent the majority of chronic diseases, indicating a great need for prevention programmes and awareness campaigns.[14,15]

Global recommendations for physical activity (PA) stipulate a daily minimum of 60 minutes of moderate-to-vigorous PA for children between 5 and 17 years old. Walter and colleagues showed that the levels of in-school PA among children from marginalized communities in South Africa do not meet the minimal requirements,[16] which was later confirmed by The Healthy Active Kids South Africa Report Card (2018).[17] Considering the above, there is an urgent need to promote PA amongst at-risk communities in South Africa. Several school-based lifestyle interventions have proved effective in increasing levels of PA and reducing obesity in children from different contexts.[18-20] However, very few school-based and large-scale PA intervention studies have been conducted in South Africa. The first study, *HealthKick*, was set in disadvantaged low-income communities in Cape Town and aimed at promoting healthy eating habits and regular participation in PA in children to reduce the risk of chronic diseases.[21] Study results were not completely satisfactory and highlighted that educators play a key role in implementing lifestyle interventions in the schools.[22,23] Some studies have reported beneficial effects of integrated community-based interventions involving the participation of teachers and children.[24-26]

The Disease, Activity and Schoolchildren's Health (*DASH*) study documented the poor health status and double burden from communicable and non-communicable diseases of children in disadvantaged communities in Port Elizabeth, South Africa.[27] It also revealed the potential for improvement through PA and health literacy interventions, following the principles that PA is key in promoting health and well-being among school children, that learning this at a young age will have long-lasting effects throughout life, and that PA is especially relevant for children from low socio-economic communities living in LMICs. Moreover, it emphasized the role of teachers

For peer review only - http://bmjopen.bmj.com/site/about/guidelines.xhtml

as influential and willing advocates.[28] Building on these results, the same research group developed *KaziBantu*, a comprehensive school-based lifestyle intervention programme aiming at promoting health literacy for both children and teachers of primary schools in disadvantaged communities in Nelson Mandela Bay (NMB).[29] Preliminary results show that over 50% of the assessed teachers presented risk factors for NCDs. Teachers responded positively to the intervention as they felt the programme was also focused on their health and well-being and took cognizance of the difficult work environment they faced daily.

Furthermore, strong evidence suggests that general and work-related stress increases the risk of mental ill-health (burnout, anxiety, impaired quality of sleep), as well as physical disease (coronary heart disease and musculoskeletal disease). These factors contribute to an increased risk for premature death.[30] This risk was confirmed in a large representative sample of South African educators (n = 21 307) working in public schools. High stress levels, lack of job satisfaction, and different stress-related physical illnesses (hypertension, heart disease, stomach ulcers, mental distress, tobacco, and alcohol misuse) were reported.[31]

Although evidence exists on the effectiveness of community-based lifestyle interventions in developing countries,[32] studies have also shown that positive effects tend to disappear in the long-term,[33] sometimes because programmes fail to be maintained.[34,35] In addition, there is still a scarcity of evidence regarding the feasibility of educational programmes in at-risk populations in disadvantaged school settings. We hypothesize that a comprehensive approach focusing specifically on high-risk children and teachers might increase implementation feasibility and ultimately lead to sustainable favourable health outcomes in both groups.

Objectives

The goal of this project is to determine the effectiveness, impact, and sustainability of the *KaziBantu* school-based health intervention programme in promoting long-lasting, positive lifestyle changes among children and teachers in disadvantaged primary schools in NMB, South Africa. To achieve this goal, the following objectives are set, for both children and teachers:

- To assess the effectiveness of the intervention in reducing risk factors for NCDs.
- To evaluate both programmes' impact and sustainability concerning the continuation of the achieved health benefits; institutionalization or continuation of the programme implementation within an organization; and continuation of the health promotion activities through community engagement.[36]
- To provide recommendations towards guidelines for a health promotion framework that may be translated into policy at the Eastern Cape Department of Education.

METHODS

Intervention

KaziBantu, which is a composite Swahili and isiXhosa phrase meaning «Active People», was designed as a dual approach consisting of two school-based health promotion interventions: *KaziKidz* and *KaziHealth*. *KaziKidz* addressed children's risk factors for NCDs, health behaviours, and psychosocial health through the

For peer review only

implementation of a tailored teaching material toolkit, teacher workshop sessions, and coaching aimed at adequate programme delivery. On the other hand, *KaziHealth* was designed as a work-place intervention targeting teachers' health and well-being through health risk assessments and personalized lifestyle coaching. The *KaziBantu* project was implemented as a cluster randomized controlled trial (RCT) from January to October 2019; the intervention has been described in detail elsewhere.[29]

After the conclusion of the RCT, control schools are to be provided with the tested *KaziKidz* teaching material and *KaziHealth* programme. Thereafter, the implementation of both programmes in all project schools will proceed independently under «real-world» conditions, i.e. without support from the project team. All teaching materials and other project resources are available online.[37]

Study design

The study has been conceived as a three-year follow-up (FU) of the *KaziBantu* project. It has been designed as an observational longitudinal study to investigate the long-term effects of both lifestyle interventions, *KaziKidz* and *KaziHealth*, on health parameters and behaviours of a cohort of high-risk children, as well as all teachers. The overall sustainability and effectiveness of the programme will be assessed using a mixed methods approach, including quantitative and qualitative data collection methods and analysis over a two-year period (Figure 1). Assessments of health outcomes will be performed at two time-points: FU1 and FU2, to evaluate the maintenance of healthy, active lifestyles and its effect at an individual level. A qualitative evaluation of the intervention implementation in the schools, as well as its maintenance in the schools' curriculum together with school principals and Department of Education (DoE) officials, will also take place at FU1 and FU2. Further information on the adoption and acceptance by teachers and parents will be measured at FU2.

The study does not involve a process evaluation of the intervention implementation; and thus, will not influence the effect of «real-world» conditions.

Research setting

The present study will be conducted in the eight schools selected for the *KaziBantu* project[29]. These are quintile three primary schools in the townships and Northern areas of NMB, in the Eastern Cape Province of South Africa. The quintiles of South African schools are determined through the national poverty table and are ranged on a scale from 1 (poorest) to 5 (least poor).[38] Quintile three schools represent no-fee paying schools and their communities are adversely affected by poverty and high unemployment rates.

Study population

Originally, in the *KaziBantu* project, both *KaziKidz* and *KaziHealth* interventions were conducted in four schools, whereas the other four participating schools were used as controls.[29] In total, 1 000 children from 4th to 6th grade (aged 8 to 16 years) were enrolled in the study; 500 children were allocated to the intervention and 500 to the

For peer review only - http://bmjopen.bmj.com/site/about/guidelines.xhtml

control arm. At the same time, 150 teachers were enrolled among all participating schools; of those, 75 took part in *KaziHealth,* whereas 75 served as waiting-list controls.

This study will follow-up all participating teachers from *KaziHealth* and a cohort of 361 *KaziKidz* children, who have been identified as having an increased risk for NCDs, defined as presenting at least one of the following cardiometabolic risk factors according to age-appropriate, standard classifications (Table 1):

► Overweight or obesity
► Pre- or hypertension
► Pre- or diabetes
► Borderline or dyslipidaemia

Data collection

Primary outcomes include anthropometric and clinical examinations, self-reported and objectively measured physical activity, self-reported diet, and self-reported psychosocial health, while secondary outcomes include age, sex, socio-economic status (SES), and school.

Standard procedures to assess the intervention's health effects on markers for NCD risk factors will be conducted by well-trained professional staff (e.g. biokineticists), who will adhere to standardized, quality-controlled protocols established during previous work of the research group in the area.

Anthropometric measurements: Body weight will be measured on a digital weighing scale and body height against a stadiometer with back erect and shoulders relaxed; body mass index (BMI) will be calculated as weight/height^2. Waist circumference will be measured with a tape between the rib cage and the iliac crest and hip circumference at the maximal circumference of the buttocks; waist-to-hip ratio will be calculated as waist circumference/hip circumference. Body fat percentage will be assessed via bioelectrical impedance analysis (BIA) with a wireless body composition monitor (Tanita MC-580; Tanita Corp., Tokyo, Japan) in participants at least 3 hours after food consumption, with an empty bladder and wearing light sports clothes.

Clinical examinations: A physical examination conducted by a healthcare professional will check for signs of anaemia, abdominal conditions, and pulmonary hypertension in children. Additionally, health status will be self-reported for intestinal symptoms, such as abdominal pain and changes in bowel movements in children. Blood pressure will be measured three times after a 5 minute-seated position with the Omron® digital blood pressure monitor and appropriate cuffs sized 17 to 22 cm for children and 22 to 32 or 32 to 42 cm for adults, which will be wrapped around the left arm with the bottom of the cuff placed about 4 cm above the elbow and the palm facing up; the blood pressure will be estimated as the average value of the second and third measurement. A full blood lipid profile will assess total cholesterol, low-density lipoprotein cholesterol (LDL-C), high-density lipoprotein cholesterol (HDL-C), triglycerides, non-HDL cholesterol (non-HDL), and cholesterol to high-density lipoprotein ratio (C-HDL ratio) with the Alere Afinion AS 100 Analyzer® point-of-care testing instrument. Glycated haemoglobin (HbA1c) will be measured from the second

For peer review only

of two blood drops extracted from the fingertip previously cleaned with alcohol and pricked with a safety lancet, using the Alere Afinion AS 100 Analyzer®. Haemoglobin concentration will be assessed for teachers with the HemoCue Hb 301 system.

Objective measurement of physical behaviours: PA will be measured for each participant by wearing an ActiGraph wGT3X-BT® accelerometer around the hip, at all times, for 7 days (5 weekdays and 2 weekend days) except for activities involving water contact.

Questionnaires: Information about PA during school hours and in their free time will be self-reported by children using adjusted questions from the PA Questionnaire for Children (PAQ-C).[39] Dietary behaviours and nutrition will be self-reported by children and teachers through a 24-hour dietary recall questionnaire.[40,41] The validated KIDSCREEN-10 questionnaire will be implemented to determine children's physical and psychological well-being.[42,43] Where necessary, questionnaires will be translated into the home language of the children, isiXhosa or Afrikaans, and additionally explained orally by students of the Nelson Mandela University. Self-reported psychosocial health in teachers will be assessed through general perceived stress [four-item Perceived Stress Scale (PSS4)],[44] work-related stress [Effort-Reward Imbalance (ERI)],[45] work and family conflict [Work and Family Conflict Scale (WAFCS)],[46] and burnout symptoms (Shirom-Melamed Burnout Measure (SMBM)[47]] questionnaires. Information about teachers' work attendance will be obtained from the schools' attendance registers.

The assessment of the interventional programme sustainability will be strengthened by qualitative research, which will follow the qualitative criteria of credibility, transferability, dependability, confirmability, and authenticity.

Interviews: Information on the implementation status of the intervention in the schools and the Eastern Cape school curricula, as well as the strengths, hurdles, and prospects of its implementation and maintenance, will be collected via semi-structured interviews organized with the principals of all participating schools, as well as with authorities from the Eastern Cape Department of Education, the Provincial Teacher Development Institute and the Nelson Mandela University. The interview questions are self-designed and will be piloted with two representatives of typical participants to ensure their validity. Thereafter, questions will be adapted for the final study interviews.

Focus groups: Information about community feasibility and acceptance of both *KaziKidz* and *KaziHealth* will be gathered during 16 focus group discussion (FGD) sessions of 10 participants each, organized with groups of parents and teachers from all participating schools. Groups of parents and teachers will be assembled separately to assess settings, both at home and in school. An equal FGD participation within the groups will be observed.

Data management

Throughout the study, personal data will be anonymised through subject identification numbers. All data will be saved on SWITCHdrive, a non-commercial cloud data storage service for institutions of higher education in Switzerland. Stored data will be

For peer review only - http://bmjopen.bmj.com/site/about/guidelines.xhtml

backed up weekly and the principal investigator will supervise the content to ensure high data quality. Data collected will be accessible only to authorized personnel (investigators and, potentially, ethics committee members) and exclusively used for scientific research. Quantitative data will be double-entered and validated in EpiData 3.1 and once cleaned, transferred to STATA for statistical analysis. Interviews and FGDs will be audio-recorded, transcribed, and coded. At the end of the study, data will be shared using the publicly accessible data repository Zenodo.

Data analysis

Anthropometric, clinical indicators and objectively measured PA will be characterized by means and standard deviations, if they are close to normal distribution, and otherwise by medians and interquartile ranges. Data on self-reported health status, PA, and dietary patterns collected in questionnaires will be treated as categorical and described by their frequency distributions. Data analysis from interviews and FGDs will focus on content analysis[48] and an inductive approach based on Grounded Theory.[49] In subsequent rounds of analysis, each transcript will be worked through systematically and responses "cross-coded" to the locations where they fit thematically. Inter-linkages will then be explored, and apparently contradicting statements juxtaposed with more widely held views. Next, a series of proposition statements will be developed and used to structure the findings. Data analysis of FGDs will take into consideration the influence of power inequalities between social groups (e.g. older vs. younger, men vs. women) to guard against a potential bias in the results.

Cross-sectional analysis

Associations of health and PA outcomes with personal characteristics (e.g. gender, SES) will be assessed post-intervention, i.e. from data collected after the conclusion of the RCT. It will be adjusted for the type of school (i.e. intervention vs. control) and potential clustering within schools and classes, respective random intercepts will be used.

Mixed linear models: For continuous outcomes.

Mixed logistic regression: For categorical outcome measures, after suitable dichotomization of polytomous outcomes.

Longitudinal analysis

Mixed linear regression: Associations between longitudinal changes in continuous variables (e.g. BMI) and individual characteristics, like gender and SES, will be assessed with random intercepts for schools and classes. Longitudinal changes in these outcomes will also be compared between the previous intervention and control schools after adjustment for personal characteristics. Moreover, changes in health parameters will be regressed against changes in PA and nutritional parameters. All analyses will be conducted, with and without adjustment, for the post-intervention values of the respective outcomes. Also, potential associations between adjusted school-specific means of longitudinal changes in health parameters and the retrospectively assessed degrees of implementation of intervention measures in the

For peer review only - http://bmjopen.bmj.com/site/about/guidelines.xhtml

respective schools will be assessed.

Mixed logistic regression: Longitudinal changes in categorical outcomes will be analysed by dichotomizing polytomous outcomes. Analyses will be stratified according to the post-intervention value of the dichotomized outcome, using mixed logistic regression models of the follow-up outcome with the same random effects structure and the same predictor variables, as previously described, for the mixed linear models. Additional analyses will be conducted by treating the post-intervention and the two consequent FU outcomes as repeated measures.

To adjust for biases associated with missing data and/or loss-to-follow-up, inverse probability weighting will be used. Data imputation will be considered as an additional option if the drop-out rate is moderate (i.e. < 20%).

Impact analysis

For the second objective of assessing the overall community impact of the intervention, its acceptability, adoption, implementation, and maintenance will be explored at the individual, community, and organizational level. For this purpose, a systematic analysis will be performed based on the RE-AIM evaluation framework and its proposed dimensions (Reach, Efficacy, Adoption, Implementation, and Maintenance).[50] Each dimension will be analysed separately, using pieces of data collected by the above mentioned methods and as described in Table 2, and results will be expressed as percentages. Ultimately, the combination of all five dimensions will result in a score that Glasgow et al. refer to as "public health impact" or "population-based effect".

Participant and Public Involvement

Adult participants were involved in the conception of the research question by identifying their pivotal role in both the implementation and maintenance of the intervention. They will collaborate in the methodology via interviews and FGDs, where preliminary results will be presented for their verification. Participants will also contribute to the results by reporting on the burden of the intervention and specific local needs.

Ethics and dissemination

The proposed research will be carried out according to the international ethical, scientific and clinical practice standards established in the Declaration of Helsinki, gathered in the International Conference on Harmonization (ICH) Guidelines, and encouraged by the World Health Organization (WHO). Ethical approval has been granted by the Nelson Mandela University Research Ethics Committee (ref. no. H20-HEA-HMS-001) and the Ethics Committee Northwest and Central Switzerland (EKNZ; ref. no. Req-2020-00430). Furthermore, the study has been registered in ISRCTN (reference number ISRCTN15648510) a primary registry in the WHO International Clinical Trials Registry Platform (ICTRP). Before taking part in any further assessment, as proposed by this study, participants will be informed about the study, alongside their right to voluntary participation and withdrawal at any time, without justification. Oral assent will be sought from children and written consent from

For peer review only - http://bmjopen.bmj.com/site/about/guidelines.xhtml

their guardians, as well as from the participating teachers.

Study results will be summarized in manuscripts and submitted for publication to open-access, peer-reviewed scientific journals. Results will also be presented at relevant national and international meetings and workshops.

Methodological limitations

Any participating school may decide not to implement the intervention after completion of the *KaziBantu* trial. This will be assessed during the key informant interviews, in which case, the researchers will offer additional support through educational workshops or teacher coaching. However, if further implementation of the programme is still undesired, it will be aimed at measuring the corresponding children and teachers at the foreseen follow-up time-points. Since the studied cohort does not involve the follow-up of a control group, participants from schools refusing the implementation would serve as comparison groups for potential health and behavioural effects. Furthermore, information about the reasons for discontinuation will be sought and this information will be considered for the feasibility assessment.

The coronavirus (Covid-19) pandemic is posing severe constraints on the South African health and education systems. Currently, it is difficult to foresee the development of the pandemic and the extent of its consequences both for the South African society and specifically for the continuation of our intervention in the Eastern Cape schools. As this protocol was launched and designed before the Covid-19 crisis and due to the uncertainty about the feasibility of performing the planned measurements, the study design and assessment time points presented here are subject to change.

DISCUSSION

A total of 41 million deaths per year worldwide are attributable to non-communicable, chronic conditions, such as diabetes, coronary heart diseases, or cancer. Of those deaths, it is estimated that around 80% are preventable because they are associated with poor diets, physical inactivity, alcohol addiction, tobacco use, and polluted environments. However, NCD aetiology is very complex and individuals cannot be held responsible because they live in a system that encourages unhealthy behaviours. Hence, aiming merely to change behaviours might be complicated and ineffective over time. Instead, the focus should be on tailoring interventions aimed at creating environments that promote healthy lifestyles, making the system more beneficial, accessible, and affordable for everyone. This is especially important in the early stages of life, where healthy behaviours can be adopted naturally and maintained throughout life. Furthermore, NCDs are a major threat to individual and national economic development: unhealthy habits acquired during childhood will most likely lead to unhealthy adults who are unable to develop to their full potential.[51]

The complexity of lifestyle interventions, such as *KaziKidz* and *KaziHealth,* and their successful implementation reside in their multi-dimensional nature, that is, the involvement of individual, environmental and policy factors that interact at multiple social levels (individual, community, and organizational institutional). The RE-AIM framework will be followed to predict the feasibility of both interventions in terms of

effectiveness, addressing whole populations, being long-lasting, and becoming institutionalized.[50,52,53] The information obtained from linking the intervention integration level in the schools with the personal adoption of behavioural changes and the achieved health effects both in children and teachers, will yield unprecedented results on lifestyle interventions' potential to effectively promote the acquisition and maintenance of healthy lifestyles during childhood and adulthood that will ultimately improve overall community health and well-being.

Furthermore, due to the multi-factorial nature of NCDs, collaboration among different sectors, such as governments, communities, academia, and the private sector, is necessary. The comprehensive evaluation of a tailored school-based health promotion intervention presented in this paper could provide unique evidence and support the dissemination of the intervention and collaboration between partners from different sectors,[54] ultimately influencing local policy.[55]

TABLES AND FIGURES

Table 1 Classification criteria for cardiometabolic risk in children

Risk factor	Cut-off points
**Adiposity**[56,57]	**BMI** (SD)
Overweight	>+1 and ≤+2
Obesity	>+2
**Blood pressure**[58,59]	**SBP and/or DBP** (percentiles and mmHg)
Elevated blood pressure	≥90th and <95th or <90th but ≥120/80
Hypertension stage 1	≥95th and ≤99th or ≥130/80
Hypertension stage 2	>99th or ≥140/90
**Glycaemia**[60]	**HbA1c** (mmol/mol)
Pre-diabetes	>39 and <48
Diabetes	≥48
**Dyslipidaemia**[61,62]	**TC** (mmol/l)
Borderline	≥4.40 and 5.15
Dyslipidaemia	≥5.15

BMI: body mass index; SD: standard deviation; SBP: systolic blood pressure; DBP: diastolic blood pressure; HbA1c: Glycated haemoglobin; TC: total cholesterol.

Table 2 Impact analysis evaluation based on the RE-AIM framework.

Dimensions	Social level	Evaluation criteria
**Reach**<br>Proportion of the target population that has participated in the intervention	**Individual**<br>Children<br>Teachers	Number and characteristics of children who:<br>➢ Have received at least half the planned lessons, as reported by the teachers

For peer review only - http://bmjopen.bmj.com/site/about/guidelines.xhtml

		Number and characteristics of teachers who:<br>➢ Have received the health risk assessment<br>➢ Have attended at least one face-to-face coaching session
**Efficacy**<br>Success rate of the intervention in reducing risk factors	**Individual**<br>Children<br>Teachers	Changes in:<br>➢ Anthropometric measurements<br>➢ Clinical parameters<br>➢ Quality of life
**Adoption**<br>Proportion of settings that has incorporated the intervention	**Organizational**<br>School teachers<br>School principals<br><br>**Community**<br>School teachers<br>Parents	Number and characteristics of:<br>➢ Schools that implemented the intervention<br>➢ Health-promoting activities per school<br><br>Number and characteristics of:<br>➢ Teachers who have adopted *KaziHealth* recommendations<br>➢ Parents aware of and supporting *KaziKidz* activities
**Implementation**<br>Extent to which the intervention has been embedded in the schools under naturalistic conditions	**Organizational**<br>School principals	Number of:<br>➢ Grades and classes participating in the intervention<br>➢ Terms per school year in which the intervention is being taught<br>➢ Staff involved in delivering the intervention
**Maintenance**<br>Adherence to and dissemination of the intervention	**Individual**<br>Children<br>Teachers<br><br>**Organizational**<br>Department of Education<br>Nelson Mandela University	Changes in:<br>➢ Physical activity and dietary behaviour<br><br>Number and characteristics of:<br>➢ Schools/districts that have the intervention integrated into the curriculum/environment<br>➢ Actions taken towards its permanent integration (policy), e.g. teaching agenda of future teachers

Acknowledgments

The study group would like to thank Dr Bruce Damons (Director of Centre for the Community School, Nelson Mandela University, Port Elizabeth) for acting as an

For peer review only

advisor on the project and being a key intermediary between the universities and the schools. We would also like to thank PD Dr Christian Schindler, who provided input for the statistical analysis of the study. This study takes place under the umbrella of the UNESCO Chair on “Physical Activity and Health in Educational Settings”.

Contributors

PA, LA, and IM conceptualised the study and led the development of the general project. MG, UP, CW, RdR, PS, JU, MB, and HS contributed to the development of the study design, methodology, and data analysis plan. PA and LA drafted this manuscript, PA elaborated the *KaziKidz*’s part, while LA contributed with the *KaziHealth*’s fraction. All co-authors commented on revised drafts. All authors approved the final version of the manuscript.

Data sharing

Individual de-identified participant data will be shared using the publicly accessible data repository Zenodo. Data will become available at the end of the study for at least 20 years.

Funding

This study is funded by the Swiss National Science Foundation (SNF; Bern, Switzerland; project no. 320030_192651)

Competing interests

All authors have completed the ICMJE uniform disclosure form at www.icmje.org/coi_disclosure.pdf and declare: PA and UP had financial support from the SNF for the submitted work; no financial relationships with any organizations that might have an interest in the submitted work in the previous three years; no other relationships or activities that could appear to have influenced the submitted work.

Provenance and peer review

Not commissioned; externally peer-reviewed.

For peer review only

REFERENCES

1. Blair DM, Stewart-Brown PS, Waterston DT, Crowther DR. Child Public Health, 2010.
2. Steinmann P, Usubalieva J, Imanalieva C, et al. Rapid appraisal of human intestinal helminth infections among schoolchildren in Osh oblast, Kyrgyzstan. *Acta Trop* 2010;116(3):178-84.
3. Victora CG, Adair L, Fall C, et al. Maternal and child undernutrition: consequences for adult health and human capital. *The Lancet* 2008;371(9609):340-57.
4. Dewey KG, Begum K. Long-term consequences of stunting in early life. *Matern Child Nutr* 2011;7 Suppl 3:5-18.
5. Collaboration NCDRF. Rising rural body-mass index is the main driver of the global obesity epidemic in adults. *Nature* 2019;569(7755):260-64.
6. Gouda HN, Charlson F, Sorsdahl K, et al. Burden of non-communicable diseases in sub-Saharan Africa, 1990–2017: results from the Global Burden of Disease Study 2017. *The Lancet Global Health* 2019;7(10):e1375-e87.
7. Lozano R, Naghavi M, Foreman K, et al. Global and regional mortality from 235 causes of death for 20 age groups in 1990 and 2010: a systematic analysis for the Global Burden of Disease Study 2010. *The Lancet* 2012;380(9859):2095-128.
8. Lim SS, Vos T, Flaxman AD, et al. A comparative risk assessment of burden of disease and injury attributable to 67 risk factors and risk factor clusters in 21 regions, 1990–2010: a systematic analysis for the Global Burden of Disease Study 2010. *The Lancet* 2012;380(9859):2224-60.
9. Murray CJL, Vos T, Lozano R, et al. Disability-adjusted life years (DALYs) for 291 diseases and injuries in 21 regions, 1990–2010: a systematic analysis for the Global Burden of Disease Study 2010. *The Lancet* 2012;380(9859):2197-223.
10. Steyn NP, McHiza ZJ. Obesity and the nutrition transition in Sub-Saharan Africa. *Ann N Y Acad Sci* 2014;1311:88-101.
11. Moselakgom VK, Staden MV. Physical Activity and Risk Factors of Cardio-metabolic Diseases in South African Children. *Asian Journal of Scientific Research* 2018;11(4):498-505.
12. Berenson GS, Srinivasan SR, Bao W, et al. Association between Multiple Cardiovascular Risk Factors and Atherosclerosis in Children and Young Adults. *New England Journal of Medicine* 1998;338(23):1650-56.
13. Joshi SM, Katre PA, Kumaran K, et al. Tracking of cardiovascular risk factors from childhood to young adulthood — the Pune Children's Study. *International Journal of Cardiology* 2014;175(1):176-78.
14. Yawson AE, Abuosi AA, Badasu DM, et al. Non-communicable diseases among children in Ghana: health and social concerns of parent/caregivers. *Afr Health Sci* 2016;16(2):378-88.
15. WHO. Global Strategy on Diet, Physical Activity and Health. 2004
16. Walter CM. In-school physical activity patterns of primary school learners from disadvantaged schools in South Africa. *African Journal for Physical, Health Education, Recreation and Dance (AJPHERD)* 2011;17(4):780-89.
17. Draper CE. Results from the Healthy Active Kids South Africa
2018 Report Card. *S Afr J Child Health* 2018;13(3):130-36.
18. Puder JJ, Marques-Vidal P, Schindler C, et al. Effect of multidimensional lifestyle intervention on fitness and adiposity in predominantly migrant

preschool children (Ballabeina): cluster randomised controlled trial. *BMJ* 2011;343:d6195.
19. Wilson DK, Van Horn ML, Kitzman-Ulrich H, et al. Results of the "Active by Choice Today" (ACT) randomized trial for increasing physical activity in low-income and minority adolescents. *Health Psychol* 2011;30(4):463-71.
20. Lambrinou CP, Androutsos O, Karaglani E, et al. Effective strategies for childhood obesity prevention via school based, family involved interventions: a critical review for the development of the Feel4Diabetes-study school based component. *BMC Endocr Disord* 2020;20(Suppl 2):52.
21. Draper CE, de Villiers A, Lambert EV, et al. HealthKick: a nutrition and physical activity intervention for primary schools in low-income settings. *BMC Public Health* 2010;10(1):398.
22. de Villiers A, Steyn NP, Draper CE, et al. Implementation of the HealthKick intervention in primary schools in low-income settings in the Western Cape Province, South Africa: a process evaluation. *BMC Public Health* 2015;15:818.
23. Blaine RE, Franckle RL, Ganter C, et al. Using School Staff Members to Implement a Childhood Obesity Prevention Intervention in Low-Income School Districts: the Massachusetts Childhood Obesity Research Demonstration (MA-CORD Project), 2012-2014. *Prev Chronic Dis* 2017;14:E03.
24. Gorely T, Nevill ME, Morris JG, Stensel DJ, Nevill A. Effect of a school-based intervention to promote healthy lifestyles in 7-11 year old children. *Int J Behav Nutr Phys Act* 2009;6:5.
25. Rosario R, Oliveira B, Araujo A, et al. The impact of an intervention taught by trained teachers on childhood overweight. *Int J Environ Res Public Health* 2012;9(4):1355-67.
26. Innerd AL, Azevedo LB, Batterham AM. The effect of a curriculum-based physical activity intervention on accelerometer-assessed physical activity in schoolchildren: A non-randomised mixed methods controlled before-and-after study. *PLoS One* 2019;14(12):e0225997.
27. Gerber M, Müller I, Walter C, et al. Physical activity and dual disease burden among South African primary schoolchildren from disadvantaged neighbourhoods. *Prev Med* 2018;112:104-10.
28. Müller I, Schindler C, Adams L, et al. Effect of a Multidimensional Physical Activity Intervention on Body Mass Index, Skinfolds and Fitness in South African Children: Results from a Cluster-Randomised Controlled Trial. *Int J Environ Res Public Health* 2019;16(2)
29. Müller I, Smith D, Adams L, et al. Effects of a School-Based Health Intervention Program in Marginalized Communities of Port Elizabeth, South Africa (the KaziBantu Study): Protocol for a Randomized Controlled Trial. *JMIR Res Protoc* 2019;8(7):e14097.
30. Kivimäki M, Leino-Arjas P, Luukkonen R, et al. Work stress and risk of cardiovascular mortality: prospective cohort study of industrial employees. *BMJ* 2002;325(7369):857.
31. Peltzer K, Shisana O, Zuma K, Van Wyk B, Zungu-Dirwayi N. Job stress, job satisfaction and stress-related illnesses among South African educators. *Stress and Health* 2009;25(3):247-57.
32. Sarrafzadegan N, Kelishadi R, Esmaillzadeh A, et al. Do lifestyle interventions work in developing countries? Findings from the Isfahan Healthy Heart Program in the Islamic Republic of Iran. *Bull World Health Organ* 2009;87(1):39-50.
33. Gerber M, Fuchs R, Pühse U. Follow-up of a short motivational and volitional exercise-intervention trial with overweight and obese individuals. *Schweizerische Zeitschrift für «Sportmedizin und Sporttraumatologie»* 2010;58(4):108–14.
34. Sarrafzadegan N, Rabiei K, Wong F, et al. The sustainability of interventions of a community-based trial on children and adolescents' healthy lifestyle. *ARYA Atheroscler* 2014;10(2):107-17.
35. Nguyen S, Hacker AL, Henderson M, et al. Physical Activity Programs with Post-Intervention Follow-Up in Children: A Comprehensive Review According to Categories of Intervention. *Int J Environ Res Public Health* 2016;13(7)
36. Shediac-Rizkallah MC, Bone LR. Planning for the sustainability of community-based health programs: conceptual frameworks and future directions for research, practice and policy. *Health Education Research* 1998;13(1):87-108.
37. UNESCO Chair on Physical Activity and Health in Educational Settings. KaziBantu project and teaching materials. 2019; Retrieved 13 September 2020; https://kazibantu.org/.
38. Chutgar AK, A. . School money funding flaws. *Human Science Research Council Review* 2009;7(4):18 - 19.
39. Kowalski KC, Crocker PRE, Donen RM. The Physical Activity Questionnaire for Older Children (PAQ-C) and Adolescents (PAQ-A) Manual. Saskatchewan Uo. 2004.
40. Shim JS, Oh K, Kim HC. Dietary assessment methods in epidemiologic studies. *Epidemiol Health* 2014;36:e2014009.
41. Raymond LKMJL. Krause's Food & the Nutrition Care Process. 14th ed. Elsevier - Health Sciences Division 2016.
42. The European KIDSCREEN Group. The KIDSCREEN-10 Index: Health-Related Quality of Life Questionnaire for Children and Adolescents aged from 8 to 18 years 2011; Retrieved 13 September 2020; https://www.kidscreen.org/english/questionnaires/kidscreen-10-index/.
43. Ravens-Sieberer U, Erhart M, Rajmil L, et al. Reliability, construct and criterion validity of the KIDSCREEN-10 score: a short measure for children and adolescents' well-being and health-related quality of life. *Qual Life Res* 2010;19(10):1487-500.
44. Reis RS, Hino AA, Anez CR. Perceived stress scale: reliability and validity study in Brazil. *J Health Psychol* 2010;15(1):107-14.
45. Zurlo MC, Pes D, Siegrist J. Validity and reliability of the effort-reward imbalance questionnaire in a sample of 673 Italian teachers. *Int Arch Occup Environ Health* 2010;83(6):665-74.
46. Pujol-Cols L. Development and validation of the Spanish Work-Family Conflict Scale (SP-WFCS): Evidence from two independent samples in Argentina. *Current Psychology* 2019
47. Ülbeğî İ, İplîk, E. The Reliability and Validity of the Shirom-Melamed Burnout Measure. *Cag University Journal of Social Sciences* 2017;14(1):19-30.
48. Hsieh HF, Shannon SE. Three approaches to qualitative content analysis. *Qual Health Res* 2005;15(9):1277-88.
49. Dahlgren L, Emmelin M, Winkvist A. Qualitative methodology for international public health. Umeå Universitet, 2007.
50. Glasgow REV, T. M.; Boles S. M. Evaluating the public health impact of health promotion interventions the RE-AIM framework. *Am J Public Health* 1999;89(9):1322-7.
51. Craigie AM, Lake AA, Kelly SA, Adamson AJ, Mathers JC. Tracking of obesity-related behaviours from childhood to adulthood: A systematic review. *Maturitas* 2011;70(3):266-84.
52. Gaglio B. The RE_AIM Framework: A Systematic Review of use over time. 2013
53. Lew MS, L'Allemand D, Meli D, et al. Evaluating a childhood obesity program with the Reach, Effectiveness, Adoption, Implementation, Maintenance (RE-AIM) framework. *Prev Med Rep* 2019;13:321-26.
54. Naylor PJ, Nettlefold L, Race D, et al. Implementation of school based physical activity interventions: a systematic review. *Prev Med* 2015;72:95-115.
55. Singh A, Bassi S, Nazar GP, et al. Impact of school policies on non-communicable disease risk factors - a systematic review. *BMC Public Health* 2017;17(1):292.
56. World Health Organization. Growth reference data for 5-19 years: BMI-for-age. 2007; Retrieved 13 September 2020; https://www.who.int/growthref/who2007_bmi_for_age/en/.
57. de Onis M, Onyango AW, Borghi E, et al. Development of a WHO growth reference for school-aged children and adolescents. *Bull World Health Organ* 2007;85(9):660-7.
58. The fourth report on the diagnosis, evaluation, and treatment of high blood pressure in children and adolescents. *Pediatrics* 2004;114(2 Suppl 4th Report):555-76.
59. Rosner B, Cook N, Portman R, Daniels S, Falkner B. Determination of blood pressure percentiles in normal-weight children: some methodological issues. *Am J Epidemiol* 2008;167(6):653-66.
60. American Diabetes A. 2. Classification and Diagnosis of Diabetes: Standards of Medical Care in Diabetes-2020. *Diabetes Care* 2020;43(Suppl 1):S14-S31.
61. National Cholesterol Education Program (NCEP): Highlights of the Report of the Expert Panel on Blood Cholesterol Levels in Children and Adolescents. *Pediatrics* 1992;89(3):495.

For peer review only - http://bmjopen.bmj.com/site/about/guidelines.xhtml

62. Expert Panel on Integrated Guidelines for Cardiovascular H, Risk Reduction in C, Adolescents, National Heart L, Blood I. Expert panel on integrated guidelines for cardiovascular health and risk reduction in children and adolescents: summary report. *Pediatrics* 2011;128 Suppl 5:S213-56.

For peer review only

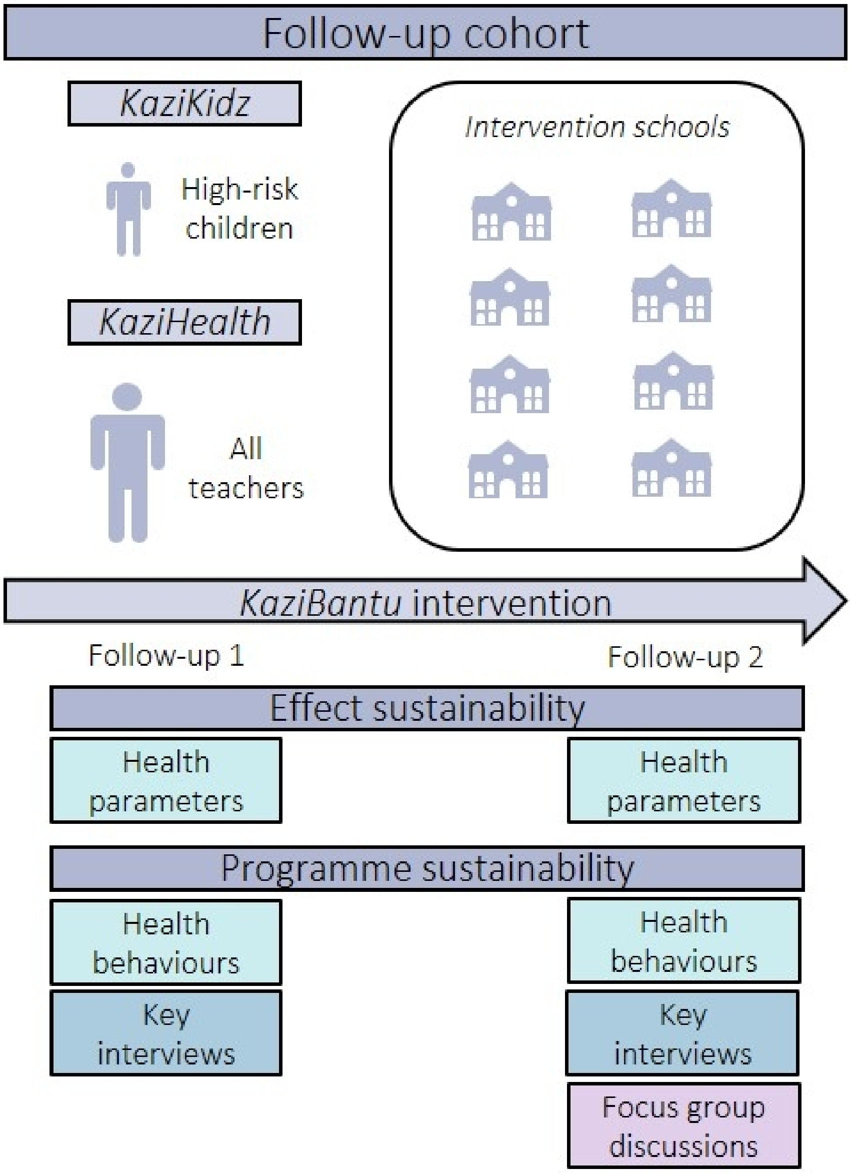

Figure 1 Overview of the follow-up study design to assess the sustainability and effectiveness of KaziKidz/KaziHealth.

80x114mm (150 x 150 DPI)

STROBE Statement—Checklist of items that should be included in reports of ***cohort studies***

	Item No	Recommendation	Page No
**Title and abstract**	1	(*a*) Indicate the study's design with a commonly used term in the title or the abstract	1
		(*b*) Provide in the abstract an informative and balanced summary of what was done and what was found	2
**Introduction**			
Background/rationale	2	Explain the scientific background and rationale for the investigation being reported	3-4
Objectives	3	State specific objectives, including any prespecified hypotheses	4
**Methods**			
Study design	4	Present key elements of study design early in the paper	5
Setting	5	Describe the setting, locations, and relevant dates, including periods of recruitment, exposure, follow-up, and data collection	5
Participants	6	(*a*) Give the eligibility criteria, and the sources and methods of selection of participants. Describe methods of follow-up	5-6
		(*b*) For matched studies, give matching criteria and number of exposed and unexposed	n/a
Variables	7	Clearly define all outcomes, exposures, predictors, potential confounders, and effect modifiers. Give diagnostic criteria, if applicable	6-7
Data sources/ measurement	8*	For each variable of interest, give sources of data and details of methods of assessment (measurement). Describe comparability of assessment methods if there is more than one group	6-7
Bias	9	Describe any efforts to address potential sources of bias	9
Study size	10	Explain how the study size was arrived at	6
Quantitative variables	11	Explain how quantitative variables were handled in the analyses. If applicable, describe which groupings were chosen and why	8-9
Statistical methods	12	(*a*) Describe all statistical methods, including those used to control for confounding	8-9
		(*b*) Describe any methods used to examine subgroups and interactions	8-9
		(*c*) Explain how missing data were addressed	9
		(*d*) If applicable, explain how loss to follow-up was addressed	9
		(*e*) Describe any sensitivity analyses	n/a
**Results**			
Participants	13*	(a) Report numbers of individuals at each stage of study—eg numbers potentially eligible, examined for eligibility, confirmed eligible, included in the study, completing follow-up, and analysed	n/a
		(b) Give reasons for non-participation at each stage	n/a
		(c) Consider use of a flow diagram	n/a
Descriptive data	14*	(a) Give characteristics of study participants (eg demographic, clinical, social) and information on exposures and potential confounders	n/a
		(b) Indicate number of participants with missing data for each variable of interest	n/a
		(c) Summarise follow-up time (eg, average and total amount)	n/a
Outcome data	15*	Report numbers of outcome events or summary measures over time	n/a

For peer review only - http://bmjopen.bmj.com/site/about/guidelines.xhtml

Main results	16	(*a*) Give unadjusted estimates and, if applicable, confounder-adjusted estimates and their precision (eg, 95% confidence interval). Make clear which confounders were adjusted for and why they were included	n/a
		(*b*) Report category boundaries when continuous variables were categorized	n/a
		(*c*) If relevant, consider translating estimates of relative risk into absolute risk for a meaningful time period	n/a
Other analyses	17	Report other analyses done—eg analyses of subgroups and interactions, and sensitivity analyses	n/a
**Discussion**			
Key results	18	Summarise key results with reference to study objectives	n/a
Limitations	19	Discuss limitations of the study, taking into account sources of potential bias or imprecision. Discuss both direction and magnitude of any potential bias	2-3, 10
Interpretation	20	Give a cautious overall interpretation of results considering objectives, limitations, multiplicity of analyses, results from similar studies, and other relevant evidence	10-11
Generalisability	21	Discuss the generalisability (external validity) of the study results	2-3, 10-11
**Other information**			
Funding	22	Give the source of funding and the role of the funders for the present study and, if applicable, for the original study on which the present article is based	13

*Give information separately for exposed and unexposed groups.

Note: An Explanation and Elaboration article discusses each checklist item and gives methodological background and published examples of transparent reporting. The STROBE checklist is best used in conjunction with this article (freely available on the Web sites of PLoS Medicine at http://www.plosmedicine.org/, Annals of Internal Medicine at http://www.annals.org/, and Epidemiology at http://www.epidem.com/). Information on the STROBE Initiative is available at http://www.strobe-statement.org.

For peer review only

For peer review only - http://bmjopen.bmj.com/site/about/guidelines.xhtml

BMJ Open

Sustainability of a school-based health intervention for prevention of non-communicable diseases in marginalised communities: protocol for a mixed methods cohort study

Journal:	*BMJ Open*
Manuscript ID	bmjopen-2020-047296.R1
Article Type:	Original research
Date Submitted by the Author:	05-May-2021
Complete List of Authors:	Arnaiz, Patricia; University of Basel, Department of Sport, Exercise and Health<br>Adams, Larissa; Nelson Mandela Metropolitan University, Department of Human Movement Science<br>Müller, Ivan; University of Basel, Department of Sport, Exercise and Health<br>Gerber, Markus; University of Basel, Department of Sport, Exercise and Health<br>Walter, Cheryl; Nelson Mandela Metropolitan University, Department of Human Movement Science<br>du Randt, Rosa; Nelson Mandela Metropolitan University, Department of Human Movement Science<br>Steinmann, Peter ; Swiss Tropical and Public Health Institute; University of Basel<br>Bergman, Manfred; University of Basel, Department of Social Sciences<br>Seelig, Harald; University of Basel, Department of Sport, Exercise and Health<br>van Greunen, Darelle; Nelson Mandela Metropolitan University, Centre for Community Technologies<br>Utzinger, Jürg; Swiss Tropical and Public Health Institute; University of Basel<br>Pühse, Uwe ; University of Basel, Department of Sport, Exercise and Health
**Primary Subject Heading**:	Public health
Secondary Subject Heading:	Sports and exercise medicine, Evidence based practice, Public health
Keywords:	PREVENTIVE MEDICINE, Community child health < PAEDIATRICS, SPORTS MEDICINE, PUBLIC HEALTH

SCHOLARONE™
Manuscripts

I, the Submitting Author has the right to grant and does grant on behalf of all authors of the Work (as defined in the below author licence), an exclusive licence and/or a non-exclusive licence for contributions from authors who are: i) UK Crown employees; ii) where BMJ has agreed a CC-BY licence shall apply, and/or iii) in accordance with the terms applicable for US Federal Government officers or employees acting as part of their official duties; on a worldwide, perpetual, irrevocable, royalty-free basis to BMJ Publishing Group Ltd ("BMJ") its licensees and where the relevant Journal is co-owned by BMJ to the co-owners of the Journal, to publish the Work in this journal and any other BMJ products and to exploit all rights, as set out in our licence.

The Submitting Author accepts and understands that any supply made under these terms is made by BMJ to the Submitting Author unless you are acting as an employee on behalf of your employer or a postgraduate student of an affiliated institution which is paying any applicable article publishing charge ("APC") for Open Access articles. Where the Submitting Author wishes to make the Work available on an Open Access basis (and intends to pay the relevant APC), the terms of reuse of such Open Access shall be governed by a Creative Commons licence – details of these licences and which Creative Commons licence will apply to this Work are set out in our licence referred to above.

Other than as permitted in any relevant BMJ Author's Self Archiving Policies, I confirm this Work has not been accepted for publication elsewhere, is not being considered for publication elsewhere and does not duplicate material already published. I confirm all authors consent to publication of this Work and authorise the granting of this licence.

For peer review only

Sustainability of a school-based health intervention for prevention of non-communicable diseases in marginalised communities: protocol for a mixed methods cohort study

Patricia Arnaiz[1*] (PhD candidate, patricia.arnaiz@unibas.ch, https://orcid.org/0000-0001-5626-3510), Larissa Adams[2*] (PhD candidate, larissa.adams@mandela.ac.za, https://orcid.org/0000-0003-1459-1916), Ivan Müller[1] (Research associate, ivan.mueller@unibas.ch, https://orcid.org/0000-0002-6397-9979), Markus Gerber[1] (Associate professor, markus.gerber@unibas.ch, https://orcid.org/0000-0001-6140-8948), Cheryl Walter[2] (Head of Department, cheryl.walter@mandela.ac.za), Rosa du Randt[2] (Emeritus professor, rosa.durandt@mandela.ac.za), Peter Steinmann[3,4] (Project leader, peter.steinmann@swisstph.ch, https://orcid.org/0000-0003-4800-3019), Manfred Max Bergman[5] (Professor, max.bergman@unibas.ch, https://orcid.org/0000-0002-4059-5833), Harald Seelig[1] (Statistician, harald.seelig@unibas.ch, https://orcid.org/0000-0002-2178-4807), Darelle van Greunen[6] (Director of Centre, darelle.vangreunen@mandela.ac.za, https://orcid.org/0000-0002-0761-713X), Jürg Utzinger[3,4] (Director of Institute, juerg.utzinger@swisstph.ch), Uwe Pühse[1] (Head of Department, uwe.puehse@unibas.ch, https://orcid.org/0000-0003-1228-316X)

[1]Department of Sport, Exercise and Health, University of Basel, 4052 Basel, Switzerland.

[2]Department of Human Movement Science, Nelson Mandela University, 6031 Port Elizabeth, South Africa.

[3]Swiss Tropical and Public Health Institute, 4002 Basel, Switzerland.

[4]University of Basel, 4001 Basel, Switzerland.

For peer review only

[5]Department of Social Sciences, University of Basel, 4051 Basel, Switzerland.
[6]Centre for Community Technologies, Nelson Mandela University, 6031 Port Elizabeth, South Africa.

Joint first authorship

* PA and LA contributed equally to this paper.

Correspondence to

Patricia Arnaiz patricia.arnaiz@unibas.ch Department of Sport, Exercise and Health, University of Basel, Villa Burckhardt, Gellertstrasse 156, 4052 Basel, Switzerland

Word count 4,274.

Number of tables and figures

Tables 1 and 2; Figure 1.

ABSTRACT

Introduction The prevalence of chronic, lifestyle-related diseases is increasing among both adults and children from low- and middle-income countries. Despite the effectiveness of community-based interventions to address this situation, the benefits thereof may disappear, long-term, due to a lack of maintenance, especially among disadvantaged and high-risk populations. The *KaziBantu* randomized controlled trial conducted in 2019 consisted of two school-based health interventions, *KaziKidz* and *KaziHealth*. The present study will evaluate the long-term effectiveness and sustainability of these two interventions in promoting positive lifestyle changes among children and teachers in disadvantaged schools in Nelson Mandela Bay, South Africa in the context of the COVID-19 pandemic.

Methods and analysis This study has an observational, longitudinal, mixed methods design. It will follow up teachers and children from the *KaziBantu* study. All 160 teachers enrolled in *KaziHealth* will be invited to participate, while the study will focus on 361 *KaziKidz* children (aged 10 to 16 years) identified as having an increased risk for non-communicable diseases. Data collection will take place 1.5 and 2 years post-intervention and includes quantitative and qualitative methods, such as anthropometric measurements, clinical assessments, questionnaires, interviews, and focus group discussions. Analyses will encompass: prevalence of health parameters;

For peer review only

descriptive frequencies of self-reported health behaviours and quality of life; the longitudinal association of these; extent of implementation; personal experiences with the programs; and an impact analysis based on the RE-AIM framework.

Discussion In settings where resources are scarce, sustainable and effective prevention programs are needed. The purpose of this protocol is to outline the design of a study to evaluate *KaziKidz* and *KaziHealth* under real-world conditions in terms of effectiveness, being long-lasting, and becoming institutionalized. We hypothesize that a mixed methods approach will increase understanding of the interventions' capacity to lead to sustainable favourable health outcomes amid challenging environments, thereby generating evidence for policy.

Trial registration number ISRCTN15648510.

STRENGTHS AND LIMITATIONS OF THIS STUDY

- The cohort of children was purposely and objectively identified based on their high-risk profile; the follow-up of personal health outcomes is of additional value.
- The mixed-methods approach, strengthened by the involvement of the community and key stakeholders, will enable a comprehensive evaluation of the programs.
- Internationally recognised measurement methods for quantitative, as well as qualitative were selected and adapted to the study setting.
- The COVID-19 pandemic might influence schools' decision to discontinue or adapt the post-*KaziBantu* intervention and the research team will assess, but not influence, its implementation.
- The study will yield context-specific results that will be closely linked to COVID-19 measures and practices adopted by the schools.

INTRODUCTION

Socioeconomic, environmental, and cultural factors exert a significant influence on children's health and well-being.[1] In impoverished communities in low- and middle-

For peer review only

income countries (LMICs), children are especially vulnerable to illness and lags in age-appropriate development, negatively affecting their life prospects.[2-4] This precarious situation has been aggravated by the COVID-19 pandemic, which has compromised millions of South African children's education, mental health and nutrition.[5] Furthermore, the disease profile of urban populations from LMICs is approaching that of high-income countries, as the proportion of deaths attributable to chronic, lifestyle-related diseases increases.[6-8] Of particular concern are changing dietary habits and sedentary lifestyle patterns,[9,10] both known to lead to metabolic and physiological changes, such as obesity and hypertension, also in young children.[11,12] Children presenting with cardiometabolic risk factors are at particularly high risk because of their predisposition to developing non-communicable diseases (NCDs) later in life.[13,14] Thus, a reduction of undesired behaviours could potentially prevent the majority of chronic diseases, indicating a great need for prevention programs and awareness campaigns.[15,16]

Global recommendations for physical activity (PA) stipulate a daily minimum of 60 minutes of moderate-to-vigorous PA for children between 5 and 17 years old. Walter and colleagues showed that the levels of in-school PA among children from marginalized communities in South Africa do not meet the minimal requirements,[17] which was later confirmed by *'The Healthy Active Kids South Africa Report Card'* (2018).[18] Therefore, there is an urgent need to promote PA amongst at-risk communities in South Africa. Several school-based lifestyle interventions have proved effective in increasing levels of PA and reducing obesity in children from different contexts.[19-21] However, very few school-based and large-scale PA intervention studies have been conducted in South Africa. The first study, *HealthKick*, was set in disadvantaged low-income communities in Cape Town and aimed at promoting healthy eating habits and regular participation in PA in children to reduce the risk of chronic diseases.[22] Study results were not completely satisfactory and highlighted that educators play a key role in implementing lifestyle interventions in schools.[23,24] Some studies have reported beneficial effects of integrated community-based interventions involving the participation of teachers and children.[25-27]

The *'Disease, Activity and Schoolchildren's Health'* (*DASH*) study documented the

For peer review only

poor health status and double burden from communicable and non-communicable diseases of children in disadvantaged communities in Port Elizabeth, South Africa.[28] It also revealed the potential for improvement through PA and health literacy interventions, following the principles that PA is key for the promotion of health and well-being among school children, and that learning this at a young age will have long-lasting effects throughout life. Moreover, it emphasized the role of teachers as influential and willing advocates.[29] Building on these results and experience, *KaziBantu* was developed, a comprehensive school-based lifestyle intervention program aiming at promoting health literacy in both children and teachers of primary schools from disadvantaged communities in Nelson Mandela Bay (NMB).[30] *KaziBantu*, which is a composite Swahili and isiXhosa phrase meaning «Active People», was designed as a dual approach consisting of two school-based health promotion interventions, *KaziKidz* for children and *KaziHealth* for teachers, and was implemented as a cluster randomized controlled trial (RCT) between January and October 2019. Preliminary results show that more than half of the assessed children (544/975) presented at least one risk factor for NCDs. Teachers responded positively to the intervention as they felt the program was also focused on their health and well-being (individualised results were communicated to the study participants) and took cognizance of the difficult work environment they faced daily.

Furthermore, evidence suggests that general and work-related stress increases the risk of mental ill-health (burnout, anxiety, impaired quality of sleep), as well as physical disease (coronary heart disease and musculoskeletal disease). These factors seem to contribute to an increased risk of premature death.[31] This risk was confirmed in a large representative sample of South African educators (N=21,307) working in public schools. High stress levels, lack of job satisfaction, and different stress-related physical illnesses (hypertension, heart disease, stomach ulcers, mental distress, tobacco, and alcohol misuse) were reported.[32]

Although evidence exists on the effectiveness of community-based lifestyle interventions in low- and middle-income countries,[33] studies have also shown that positive effects tend to disappear in the long-term,[34] sometimes because programs fail to be maintained.[35,36] In addition, there is still a scarcity of evidence regarding the feasibility of educational programs in at-risk populations in disadvantaged school

For peer review only

settings. We hypothesize that a comprehensive approach focusing specifically on high-risk children and their teachers might increase implementation feasibility and ultimately lead to sustainable favourable health outcomes in both groups.

Objectives

The goal of this follow-up study is to determine the long-term post-RCT effectiveness, adoption and continuity of both *KaziKidz* and *KaziHealth* school-based health interventions in disadvantaged primary schools in NMB, South Africa, under real-world conditions and amid the COVID-19 pandemic. To achieve this goal, the following objectives are set:

i. To assess the effectiveness of the interventions in promoting long-lasting, positive lifestyle changes among children and teachers, thereby reducing risk factors for NCDs;
ii. To evaluate the post-intervention sustainability concerning the continuation of the program implementation in the schools as well as the institutionalization within the Eastern Cape Department of Education (ECDoE) in South Africa; and
iii. To provide recommendations towards guidelines for a health promotion framework, as well as its adaption to new contexts that may be translated into policy and practice.

METHODS

Research setting

The present study is conducted in the eight schools originally selected for the *KaziBantu* RCT[30]. These are quintile three primary schools in the townships and Northern areas of NMB, in the Eastern Cape province of South Africa. The quintiles of South African schools are determined through the national poverty table and are ranged on a scale from 1 (poorest) to 5 (least poor). Quintile three schools represent no-fee paying schools and their communities are adversely affected by poverty and high unemployment rates.

Study population

For peer review only

Originally, in the *KaziBantu* project, both *KaziKidz* and *KaziHealth* interventions were conducted in four schools, whereas the other four participating schools were used as controls.[30] In total, 975 children from 4th to 6th grade (aged 8 to 16 years) were enrolled in the study; 482 children were allocated to the intervention and 493 to the control arm. At the same time, 160 teachers were enrolled among all participating schools; of those, 85 took part in *KaziHealth,* whereas 75 served as waiting-list controls.

This study will follow up children and teachers from the *KaziBantu* trial. While all teachers who took part in *KaziHealth* have been invited to participate in this follow-up phase, a cohort of children from the *KaziKidz* intervention, who have been identified as having an increased risk for NCDs, have been included in this study. We have defined increased risk as a child presenting at least one of the following cardiometabolic risk factors: [i] overweight or obesity, [ii] elevated blood pressure or hypertension, [iii] pre- or diabetes, [iv] borderline or dyslipidaemia; according to age-appropriate, standard classifications (Table 1) and based on *KaziBantu* baseline data from July 2019. Of the 975 children who participated in *KaziKidz*, 544 were classified as «high risk» according to the above-mentioned definition. Since then, 183 children have graduated from primary school resulting in 361 eligible learners. Of those, 240 have consented to participate and have been enrolled in this follow-up study at the time this protocol was published. Of the 160 teachers enrolled in the *KaziHealth* program in 2019, 105 have consented to participate and have been enrolled in the present study so far.

Intervention

The *KaziKidz* intervention consists of a tailored, ready-made teaching material toolkit for grades 1 to 7 and addresses children's risk factors for NCDs, health behaviours, and psychosocial health. On the other hand, *KaziHealth* is designed as a workplace intervention targeting teachers' health and well-being through health risk assessments and personalized lifestyle coaching. The interventions have been described in detail elsewhere[30] and all teaching materials and other project resources are freely available online.[37]

After conclusion of the *KaziBantu* RCT in 2019, all eight schools were given the

freedom to conduct *KaziKidz* and *KaziHealth*. Thus, all children from grades 1 to 7 may have participated in *KaziKidz*, regardless of health condition. However, the implementation of *KaziKidz* and *KaziHealth* has proceeded independently and without support from the project team in all schools, under so-called «real-world» conditions. Therefore, the delivery of the programs might have been only partially implemented. For instance, some schools might have needed to adapt *KaziKidz* to comply with COVID-19-related requirements, while other schools might have not been able to, or decided not to, continue with the intervention at all.

Study design

This study represents an observational follow-up of the *KaziBantu* RCT and has been designed as a longitudinal study to investigate the long-term effects of both lifestyle interventions, *KaziKidz* and *KaziHealth*, on health parameters and behaviours of a cohort of high-risk children, as well as all teachers originally included in the RCT. The overall effectiveness and sustainability of the programs will be assessed using a mixed methods approach, including quantitative and qualitative data collection methods and analyses (Figure 1). Recruitment has started in March 2021. Assessments of health outcomes will be performed both for children and teachers at two time-points: April 2021 and September 2021, i.e. 1.5 and 2 years post-RCT, to evaluate the maintenance of healthy, active lifestyles and its effect on an individual level. As part of the post-intervention evaluation, the extent of *KaziKidz*'s implementation in the schools will be obtained from school representatives at both timepoints to be able to correlate the observed outcomes with continued participation in the original intervention. A qualitative analysis of the school headmasters' experiences with *KaziKidz,* as well as of its incorporation into the schools' curriculum with officials from the ECDoE, will take place two years post-RCT, in October 2021. Additionally, qualitative analyses will also be conducted with school teachers on their experiences with, and incorporation of, *KaziHealth* into their lifestyles two years post-RCT, in October 2021. Further information on the adoption and acceptance of *KaziKidz* by teachers and parents, and of *KaziHealth* by school headmasters' and ECDoE officials will be obtained during October 2021.

This post-intervention sustainability assessment does not involve the evaluation of

For peer review only

program delivery adequacy. Similarly, the research team will not influence the effect that «real-world» conditions might have on whether, or how, the interventions are delivered in the schools. Since the studied cohort does not involve the follow-up of a control group, participants from schools refusing the implementation would serve as comparison groups for potential health and behavioural effects.

Data collection

Primary outcomes include anthropometric and clinical examinations, self-reported and objectively measured physical activity, self-reported diet, and self-reported psychosocial health, while secondary outcomes include age, sex, socio-economic status (SES), and school. Standard procedures to assess the interventions' health effects on markers for NCD risk factors will be conducted by well-trained professional staff (e.g. biokineticists), who will adhere to standardized, quality-controlled protocols established during previous work of the research group in the area.

Anthropometric measurements: Body weight will be measured on a digital weighing scale and body height against a stadiometer with back erect and shoulders relaxed. Body mass index (BMI) will be calculated as weight (kg)/height (m)^2, and additionally, BMI-for-age stratified by sex will be determined for children according to WHO growth charts. Waist circumference will be measured with a steel tape between the rib cage and the iliac crest and hip circumference at the maximal circumference of the buttocks; waist-to-hip ratio will be calculated as waist circumference/hip circumference. Body fat percentage will be assessed via bioelectrical impedance analysis (BIA) with a wireless body composition monitor (Tanita MC-580; Tanita Corp., Tokyo, Japan) in participants at least 3 hours after food consumption.

Clinical examinations: Blood pressure will be measured three times after the participant has been seating for 5 min. The Omron® digital blood pressure monitor and appropriate cuffs sized 17 to 22 cm for children and 22 to 32 or 32 to 42 cm for adults will be used for this purpose and will be wrapped around the left arm with the bottom of the cuff placed about 4 cm above the elbow and the palm facing up. The blood pressure will be estimated as the average value of the second and third measurement. A full blood lipid profile will assess total cholesterol, low-density lipoprotein cholesterol (LDL-C), high-density lipoprotein cholesterol (HDL-C),

For peer review only

triglycerides, non-HDL cholesterol (non-HDL), and cholesterol to high-density lipoprotein ratio (C-HDL ratio) with the Alere Afinion AS 100 Analyzer® point-of-care testing instrument. Glycated haemoglobin (HbA1c) will be measured from the second of two blood drops extracted from the fingertip previously cleaned with alcohol and pricked with a safety lancet, using the Alere Afinion AS 100 Analyzer®. Haemoglobin concentration will be assessed for teachers with the HemoCue Hb 301 system.

Objective measurement of physical behaviours: PA will be measured for each participant using an accelerometer (ActiGraph wGT3X-BT®, Pensacola, Florida, USA) which will be worn around the hip, at all times, for 7 days (5 weekdays and 2 weekend days), except for activities involving water contact.

Questionnaires: Information on PA during school hours and in their free time will be self-reported by children using adjusted questions from the PA Questionnaire for Children (PAQ-C).[38] Dietary behaviours and nutrition will be self-reported by children through a tailored, validated food frequency questionnaire[39,40] and by teachers via a 24-hour dietary recall questionnaire.[41] The validated KIDSCREEN-10 questionnaire will be implemented to determine children's physical and psychological well-being.[42,43] Where necessary, questionnaires will be translated into the home language of the children, isiXhosa or Afrikaans, and additionally explained orally by students of the Nelson Mandela University. Self-reported psychosocial health in teachers will be assessed through general perceived stress [four-item Perceived Stress Scale (PSS4)],[44] work-related stress [Effort-Reward Imbalance (ERI)],[45] work and family conflict [Work and Family Conflict Scale (WAFCS)],[46] and burnout symptoms (Shirom-Melamed Burnout Measure (SMBM)[47]] questionnaires. Information about teachers' work attendance will be obtained from the schools' attendance registers. A self-developed questionnaire will be used to assess the continuity and extent of *KaziKidz*'s implementation in the schools and will be answered by the school representative.

The support for, and integration into the school curriculum of, *KaziKidz* and *KaziHealth* sustainability will be studied by qualitative research, employing credibility, transferability, dependability, confirmability, and authenticity as quality criteria when employing qualitative methods .[48]

For peer review only

Interviews: Information on the strengths, challenges, and prospects of the *KaziKidz* toolkit implementation in the schools and the Eastern Cape school curricula will be collected via semi-structured interviews. Interviewees will include principals from all participating schools (n=8), as well as representatives from the ECDoE (n=1), Provincial Teacher Development Institute (n=1), and the Nelson Mandela University (n=1). The interview schedule pertains to the perceived strengths, weaknesses, acceptance, and degree of integration of the intervention program into the school curriculum

Focus groups: Information on community feasibility and community acceptance of *KaziKidz* and *KaziHealth* will be gathered during 16 focus group discussion (FGD) sessions of 8 participants each. For *KaziKidz*, 6 focus groups will be organized and will include 24 parents and 24 teachers from previous intervention and control schools. The sessions will last approximately 60 minutes. Parents and teachers will be part of different groups to focus on the feasibility and acceptance of the program at home and in schools, respectively. For *KaziHealth*, 8 focus groups will include teachers from all schools, whereas 2 other sessions will be organized with all 8 schools' principals, on the one hand, and officials from the ECDoE, on the other.

Data management

Throughout the study, personal data will be anonymised. All data will be saved on SWITCHdrive, a Swiss non-commercial cloud data storage service for institutions of higher education. Stored data will be backed up weekly and the principal investigator will supervise the content to ensure data quality. Data collected will be accessible only to authorized personnel (investigators and ethics committee members) and exclusively used for scientific research. Quantitative data will be double-entered and validated in EpiData 3.1 and once cleaned, transferred to STATA for statistical analysis. Interviews and FGDs will be audio-recorded, transcribed, and coded. At the end of the study, data will be anonymized and shared using the publicly accessible data repository, Zenodo.

Data analysis

Anthropometric, clinical indicators and objectively measured PA will be characterized

For peer review only

by means and standard deviations, if they are close to the normal distribution, and otherwise by medians and interquartile ranges. Data on self-reported health status, PA, and dietary patterns collected in questionnaires will be treated as categorical data and described by their frequency distributions. The interview and FGD data will be analysed using qualitative content analysis,[49] and each interview and focus group transcript will be cross-coded to locations and thematic fit. To improve inter-coder reliability, two independent coders will be employed. The results of the qualitative analyses will include a taxonomy of the perceived strengths, challenges, and implementation of the intervention program.

Cross-sectional analysis

Mixed linear models: Associations of continuous health and PA outcomes with personal characteristics (e.g. sex, SES) will be assessed post-intervention, i.e. from data collected after the conclusion of the trial. It will be adjusted for the type of school (i.e. intervention vs. control) and potential clustering within schools and classes, respective random intercepts will be used.

Mixed logistic regression: For categorical outcome measures, after suitable dichotomization of polytomous outcomes, associations with the same predictor variables as for the mixed linear models will be analysed post-intervention using the same random intercepts.

Longitudinal analysis

Mixed linear regression: Associations between longitudinal changes in continuous variables (e.g. BMI) and individual characteristics, like sex and SES, will be assessed with random intercepts for schools and classes. Longitudinal changes in these outcomes will also be compared between the previous intervention and control schools after adjustment for personal characteristics. Moreover, changes in health parameters will be regressed against changes in PA and nutritional parameters. All analyses will be conducted, with and without adjustment, for the post-intervention values of the respective outcomes. Also, potential associations between adjusted school-specific means of longitudinal changes in health parameters and the retrospectively assessed degrees of implementation of intervention measures in the

For peer review only

379 respective schools will be assessed.

380 *Mixed logistic regression:* Longitudinal changes in categorical outcomes will be
381 analysed by dichotomizing polytomous outcomes. Analyses will be stratified
382 according to the post-intervention value of the dichotomized outcome, using mixed
383 logistic regression models of the follow-up outcome with the same random effects
384 structure and the same predictor variables, as previously described, for the mixed
385 linear models. Additional analyses will be conducted by treating the post-intervention
386 and the two consequent FU outcomes as repeated measures.

387 To adjust for biases associated with missing data and/or loss-to-follow-up, inverse
388 probability weighting will be used. Data imputation will be considered as an additional
389 option if the drop-out rate is moderate (i.e. < 20%).

390

391 Impact analysis

392 For the second objective of assessing the overall community impact of the
393 intervention, its acceptability, adoption, implementation, and maintenance will be
394 explored at the individual, community, and organizational level. For this purpose, a
395 systematic analysis will be performed based on the RE-AIM evaluation framework
396 and its proposed dimensions ([i] Reach, [ii] Efficacy, [iii] Adoption, [iv]
397 Implementation, and [v] Maintenance).[50] Each dimension will be analysed
398 separately, using pieces of data collected by the above-mentioned methods and as
399 described in Table 2, and results will be expressed as percentages. Ultimately, the
400 combination of all five dimensions will result in a score that Glasgow and colleagues
401 refer to as «public health impact» or «population-based effect».[50]

402

403 **Participant and public involvement**

404 Adult participants were involved in the conception of the research question by
405 identifying their pivotal role in both the implementation and maintenance of the
406 intervention. They will collaborate in the methodology via interviews and FGDs, where
407 preliminary results will be presented for their verification. Participants will also
408 contribute to the results by reporting on the burden of the interventions and specific
409 local needs.

410

For peer review only - http://bmjopen.bmj.com/site/about/guidelines.xhtml

Ethics and dissemination

The proposed research will be carried out according to the international ethical, scientific and clinical practice standards established in the Declaration of Helsinki, gathered in the International Conference on Harmonization (ICH) Guidelines, and encouraged by the WHO. Prior to study conduct, ethical approval was granted by the Nelson Mandela University Research Ethics Committee (ref. no. H20-HEA-HMS-001; 21/07/2020), the Ethics Committee Northwest and Central Switzerland (EKNZ; ref. no. Req-2020-00430; 12/05/2020) and the Eastern Cape Department of Education (07/12/2020). Furthermore, the study has been registered in ISRCTN (reference number ISRCTN15648510; 17/04/2020), a primary registry on the WHO International Clinical Trials Registry Platform (ICTRP). Participants have been informed on the study, alongside their right to voluntary participation and withdrawal at any time without justification. Before taking part in any assessment, as proposed by this study, oral assent has been sought from children and written consent from their guardians, as well as from the participating teachers.

Study results will be summarized in manuscripts and submitted for publication to open-access, peer-reviewed scientific journals. Results will also be presented at relevant national and international conferences and workshops.

Methodological limitations

The implementation of *KaziKidz* and *KaziHealth* will not be monitored by the research team and schools were given the freedom to decide whether, and to what extent, they will deliver the health interventions. Therefore, any participating school may decide not to implement the interventions after completion of the *KaziBantu* trial. The extent of *KaziKidz*'s implementation will be assessed via questionnaire with school representatives and information about the reasons for discontinuation will be sought; this information will be considered for the feasibility assessment. Similarly, the status and extent, including reasons for discontinuation, of *KaziHealth*'s implementation will also be explored and evaluated via FGDs with school teachers, headmasters' and ECDoE officials. Furthermore, for those cases where a further implementation of the interventions has not taken place, it will be aimed at measuring the corresponding children and teachers at the foreseen follow-up time-points.

For peer review only

DISCUSSION

Globally, a total of 41 million deaths are attributable to non-communicable, chronic conditions, such as diabetes, coronary heart diseases, or cancer every year. Of those deaths, it is estimated that around 80% are preventable because they are associated with poor diets, physical inactivity, alcohol addiction, tobacco use, and polluted environments. However, NCD aetiology is very complex and individuals cannot be held responsible because they live in a system that encourages unhealthy behaviours. Hence, aiming merely to change behaviours might be complicated and ineffective over time. Instead, the focus should be on tailoring interventions aimed at creating environments that promote healthy lifestyles, making the system more beneficial, accessible, and affordable for everyone. This is especially important in the early stages of life, where healthy behaviours can be adopted naturally and maintained throughout life. Furthermore, NCDs are a major threat to individual and national economic development: unhealthy habits acquired during childhood will most likely lead to unhealthy adults who are unable to develop to their full potential.[51]

The COVID-19 pandemic has highlighted the increased risk for severe disease and mortality faced by people living with chronic conditions.[52] At the same time, it has jeopardized the livelihood of millions of families, disproportionately hitting the most vulnerable and taking a special toll on children.[53] By December 2020, South African children lost up to a third of the school year, thus, not being able to complete the entire curriculum and leaving many gaps in children's education. Moreover, socioeconomic inequality is also likely to have further increased, since poorer learners and schools were least able to catch up.[54]

Upon re-opening in both 2020 and 2021, schools in South Africa have been forced to adopt strict measures to further limit the spread of COVID-19. In addition to the staggered return to the school system, the 1.5-meter social distancing requirement has led to schools incorporating what they referred to as a block teaching system, whereby schools operate at 50% or less of their capacity. More information on COVID-19-related school lockdown and closure in South Africa and the Port Elizabeth region can be found in the supplementary online material. Furthermore, the Eastern Cape Department of Basic Education advised schools not to exclude

For peer review only

any of the subjects from the curriculum, but to prioritize the more heavily weighted academic subjects, such as English, Afrikaans, Mathematics and Science. Physical education is currently not a stand-alone subject in the South African curriculum but forms part of the Life Orientation/Life Skills subject. Consequently, the likelihood of physical education receiving limited attention, and even being neglected, throughout the COVID-19 pandemic is high. Hence, we believe that the implementation of the *KaziKidz* material might have been affected too, which justifies further exploration. The complexity of lifestyle interventions, such as *KaziKidz* and *KaziHealth,* and their successful implementation reside in their multi-dimensional nature, that is, the involvement of individual, environmental and policy factors that interact at multiple social levels (individual, community, and organizational institutional). The RE-AIM framework will be followed to predict the feasibility of both interventions in terms of effectiveness, addressing whole populations, being long-lasting, and becoming institutionalized.[50,55,56] The information obtained from linking the intervention integration level in the schools with the personal adoption of behavioural changes and the achieved health effects both in children and teachers, will yield unprecedented results on lifestyle interventions' potential to effectively promote the acquisition and maintenance of healthy lifestyles during childhood and adulthood that will ultimately support overall community health and well-being. Furthermore, we believe that continuing with the assessments indicated in the methodology sections will provide valuable insights into the children's health amidst the COVID-19 pandemic, and facilitate contextually valid recommendations for future interventions, policy and practice.

Finally, due to the multi-factorial nature of NCDs, collaboration among different sectors, such as governments, communities, academia, and the private sector, is necessary. The comprehensive evaluation of a tailored school-based health promotion intervention presented in this paper could provide unique evidence and support the dissemination of the intervention and collaboration between partners from different sectors,[57] ultimately influencing local policy.[58]

TABLES AND FIGURES

Table 1 Classification criteria for cardiometabolic risk in children

Risk factor	Indicators and cut-off points
**Adiposity**[59,60]	**BMI-for-age** (SD)
Overweight	>+1 and ≤+2
Obesity	>+2
**Blood pressure**[61,62]	**SBP and/or DBP** (percentiles and mmHg)
Elevated blood pressure	≥90th and <95th or <90th but ≥120/80
Hypertension stage 1	≥95th and ≤99th or ≥130/80
Hypertension stage 2	>99th or ≥140/90
**Glycaemia**[63]	**HbA1c** (mmol/mol)
Pre-diabetes	>39 and <48
Diabetes	≥48
**Dyslipidaemia**[64,65]	**TC** (mmol/l)
Borderline	≥4.40 and 5.15
Dyslipidaemia	≥5.15

BMI: body mass index; SD: standard deviation; SBP: systolic blood pressure; DBP: diastolic blood pressure; HbA1c: glycated haemoglobin; TC: total cholesterol.

Table 2 Impact analysis evaluation based on the RE-AIM framework.

Dimensions	Social level	Evaluation criteria
**Reach**<br>Proportion of the target population that has participated in the intervention	**Individual**<br>Children<br>Teachers	Number and characteristics of children who:<br>➢ Have received at least half the planned lessons, as reported by the teachers<br>Number and characteristics of teachers who:<br>➢ Have received the health risk

For peer review only

		assessment<br>➢ Have attended at least one face-to-face coaching session
**Efficacy**<br>Success rate of the intervention in reducing risk factors	**Individual**<br>Children<br>Teachers	Changes in:<br>➢ Anthropometric measurements<br>➢ Clinical parameters<br>➢ Quality of life
**Adoption**<br>Proportion of settings that has incorporated the intervention	**Organizational**<br>School teachers<br>School principals	Number and characteristics of:<br>➢ Schools that implemented the intervention<br>➢ Health-promoting activities per school
	**Community**<br>School teachers<br>Parents	Number and characteristics of:<br>➢ Teachers who have adopted *KaziHealth* recommendations<br>➢ Parents aware of and supporting *KaziKidz* activities
**Implementation**<br>Extent to which the intervention has been embedded in the schools under naturalistic conditions	**Organizational**<br>School principals	Number of:<br>➢ Grades and classes participating in the intervention<br>➢ Terms per school year in which the intervention is being taught<br>➢ Staff involved in delivering the intervention
**Maintenance**<br>Adherence to and dissemination of the intervention	**Individual**<br>Children<br>Teachers	Changes in:<br>➢ Physical activity and dietary behaviour
	**Organizational**<br>Eastern Cape Department of	Number and characteristics of:

For peer review only

	Education Nelson Mandela University	➢ Schools/districts that have the intervention integrated into the curriculum/environment<br>➢ Actions taken towards its permanent integration (policy), e.g. teaching agenda of future teachers

Figure 1 Overview of the follow-up study design to assess the sustainability and effectiveness of *KaziKidz* and *KaziHealth*.

Acknowledgements

The research team would like to thank Dr Bruce Damons (Director of Centre for the Community School, Nelson Mandela University, Port Elizabeth) for acting as an advisor on the project and being a key intermediary between the universities and the schools. We would also like to thank PD Dr Christian Schindler, who provided input for the statistical analysis of the study. This study takes place under the auspices of the UNESCO Chair on '*Physical Activity and Health in Educational Settings*'.

Contributors

PA, LA, and IM conceptualised the study and led the development of the general project. MG, UP, CW, RdR, PS, DvG and JU contributed to the development of the study design and methodology. MB and HS provided statistical input and developed the draft data analysis plan. PA and LA drafted this manuscript, PA elaborated the *KaziKidz*'s part, while LA contributed with the *KaziHealth*'s fraction. All co-authors commented on revised drafts and approved the final version of the manuscript.

Data sharing

Individual de-identified participant data will be shared using the publicly accessible data repository Zenodo. Data will become available at the end of the study for at least 10 years.

For peer review only

Funding

This study is funded by the Swiss National Science Foundation (SNF; Bern, Switzerland; project no. 320030_192651).

Competing interests

All authors have completed the ICMJE uniform disclosure form at www.icmje.org/coi_disclosure.pdf and declare: PA and UP received financial support from the SNF for the submitted work; no financial relationships with any organizations that might have an interest in the submitted work in the previous three years; no other relationships or activities that could appear to have influenced the submitted work.

Provenance and peer review

Not commissioned; externally peer-reviewed.

REFERENCES

1. Blair DM, Stewart-Brown PS, Waterston DT, Crowther DR. Child Public Health, 2010.
2. Steinmann P, Usubalieva J, Imanalieva C, et al. Rapid appraisal of human intestinal helminth infections among schoolchildren in Osh oblast, Kyrgyzstan. *Acta Trop* 2010;116(3):178-84.
3. Victora CG, Adair L, Fall C, et al. Maternal and child undernutrition: consequences for adult health and human capital. *The Lancet* 2008;371(9609):340-57.
4. Dewey KG, Begum K. Long-term consequences of stunting in early life. *Matern Child Nutr* 2011;7 Suppl 3:5-18.
5. Zar HJ, Dawa J, Fischer GB, Castro-Rodriguez JA. Challenges of COVID-19 in children in low- and middle-income countries. *Paediatr Respir Rev* 2020;35:70-74.
6. Collaboration NCDRF. Rising rural body-mass index is the main driver of the global obesity epidemic in adults. *Nature* 2019;569(7755):260-64.
7. Gouda HN, Charlson F, Sorsdahl K, et al. Burden of non-communicable diseases in sub-Saharan Africa, 1990–2017: results from the Global Burden of Disease Study 2017. *The Lancet Global Health* 2019;7(10):e1375-e87.
8. Lozano R, Naghavi M, Foreman K, et al. Global and regional mortality from 235 causes of death for 20 age groups in 1990 and 2010: a systematic analysis for the Global Burden of Disease Study 2010. *The Lancet* 2012;380(9859):2095-128.
9. Lim SS, Vos T, Flaxman AD, et al. A comparative risk assessment of burden of disease and injury attributable to 67 risk factors and risk factor clusters in 21 regions, 1990–2010: a systematic analysis for the Global Burden of Disease Study 2010. *The Lancet* 2012;380(9859):2224-60.
10. Murray CJL, Vos T, Lozano R, et al. Disability-adjusted life years (DALYs) for 291 diseases and injuries in 21 regions, 1990–2010: a systematic analysis for the Global Burden of Disease Study 2010. *The Lancet* 2012;380(9859):2197-223.
11. Steyn NP, McHiza ZJ. Obesity and the nutrition transition in Sub-Saharan Africa. *Ann N Y Acad Sci* 2014;1311:88-101.
12. Moselakgom VK, Staden MV. Physical Activity and Risk Factors of Cardio-metabolic Diseases in South African Children. *Asian Journal of Scientific Research* 2018;11(4):498-505.
13. Berenson GS, Srinivasan SR, Bao W, et al. Association between Multiple Cardiovascular Risk Factors and Atherosclerosis in Children and Young Adults. *New England Journal of Medicine* 1998;338(23):1650-56.
14. Joshi SM, Katre PA, Kumaran K, et al. Tracking of cardiovascular risk factors from childhood to young adulthood — the Pune Children's Study. *International Journal of Cardiology* 2014;175(1):176-78.
15. Yawson AE, Abuosi AA, Badasu DM, et al. Non-communicable diseases among children in Ghana: health and social concerns of parent/caregivers. *Afr Health Sci* 2016;16(2):378-88.
16. WHO. Global Strategy on Diet, Physical Activity and Health. 2004
17. Walter CM. In-school physical activity patterns of primary school learners from disadvantaged schools in South Africa. *African Journal for Physical, Health Education, Recreation and Dance (AJPHERD)* 2011;17(4):780-89.
18. Draper CE. Results from the Healthy Active Kids South Africa
2018 Report Card. *S Afr J Child Health* 2018;13(3):130-36.
19. Puder JJ, Marques-Vidal P, Schindler C, et al. Effect of multidimensional lifestyle intervention on fitness and adiposity in predominantly migrant preschool children (Ballabeina): cluster randomised controlled trial. *BMJ* 2011;343:d6195.
20. Wilson DK, Van Horn ML, Kitzman-Ulrich H, et al. Results of the "Active by Choice Today" (ACT) randomized trial for increasing physical activity in low-income and minority adolescents. *Health Psychol* 2011;30(4):463-71.
21. Lambrinou CP, Androutsos O, Karaglani E, et al. Effective strategies for childhood obesity prevention via school based, family involved interventions: a critical review for the development of the Feel4Diabetes-study school based component. *BMC Endocr Disord* 2020;20(Suppl 2):52.
22. Draper CE, de Villiers A, Lambert EV, et al. HealthKick: a nutrition and physical activity intervention for primary schools in low-income settings. *BMC Public Health* 2010;10(1):398.
23. de Villiers A, Steyn NP, Draper CE, et al. Implementation of the HealthKick intervention in primary schools in low-income settings in the

For peer review only

Western Cape Province, South Africa: a process evaluation. *BMC Public Health* 2015;15:818.
24. Blaine RE, Franckle RL, Ganter C, et al. Using School Staff Members to Implement a Childhood Obesity Prevention Intervention in Low-Income School Districts: the Massachusetts Childhood Obesity Research Demonstration (MA-CORD Project), 2012-2014. *Prev Chronic Dis* 2017;14:E03.
25. Gorely T, Nevill ME, Morris JG, Stensel DJ, Nevill A. Effect of a school-based intervention to promote healthy lifestyles in 7-11 year old children. *Int J Behav Nutr Phys Act* 2009;6:5.
26. Rosario R, Oliveira B, Araujo A, et al. The impact of an intervention taught by trained teachers on childhood overweight. *Int J Environ Res Public Health* 2012;9(4):1355-67.
27. Innerd AL, Azevedo LB, Batterham AM. The effect of a curriculum-based physical activity intervention on accelerometer-assessed physical activity in schoolchildren: A non-randomised mixed methods controlled before-and-after study. *PLoS One* 2019;14(12):e0225997.
28. Gerber M, Müller I, Walter C, et al. Physical activity and dual disease burden among South African primary schoolchildren from disadvantaged neighbourhoods. *Prev Med* 2018;112:104-10.
29. Müller I, Schindler C, Adams L, et al. Effect of a Multidimensional Physical Activity Intervention on Body Mass Index, Skinfolds and Fitness in South African Children: Results from a Cluster-Randomised Controlled Trial. *Int J Environ Res Public Health* 2019;16(2)
30. Müller I, Smith D, Adams L, et al. Effects of a School-Based Health Intervention Program in Marginalized Communities of Port Elizabeth, South Africa (the KaziBantu Study): Protocol for a Randomized Controlled Trial. *JMIR Res Protoc* 2019;8(7):e14097.
31. Kivimäki M, Leino-Arjas P, Luukkonen R, et al. Work stress and risk of cardiovascular mortality: prospective cohort study of industrial employees. *BMJ* 2002;325(7369):857.
32. Peltzer K, Shisana O, Zuma K, Van Wyk B, Zungu-Dirwayi N. Job stress, job satisfaction and stress-related illnesses among South African educators. *Stress and Health* 2009;25(3):247-57.
33. Sarrafzadegan N, Kelishadi R, Esmaillzadeh A, et al. Do lifestyle interventions work in developing countries? Findings from the Isfahan Healthy Heart Program in the Islamic Republic of Iran. *Bull World Health Organ* 2009;87(1):39-50.
34. Gerber M, Fuchs R, Pühse U. Follow-up of a short motivational and volitional exercise-intervention trial with overweight and obese individuals. *Schweizerische Zeitschrift für «Sportmedizin und Sporttraumatologie»* 2010;58(4):108–14.
35. Sarrafzadegan N, Rabiei K, Wong F, et al. The sustainability of interventions of a community-based trial on children and adolescents' healthy lifestyle. *ARYA Atheroscler* 2014;10(2):107-17.
36. Nguyen S, Hacker AL, Henderson M, et al. Physical Activity Programs with Post-Intervention Follow-Up in Children: A Comprehensive Review According to Categories of Intervention. *Int J Environ Res Public Health* 2016;13(7)
37. UNESCO Chair on Physical Activity and Health in Educational Settings. KaziBantu project and teaching materials. 2019; Retrieved 13 September 2020; https://kazibantu.org/.
38. Kowalski KC, Crocker PRE, Donen RM. The Physical Activity Questionnaire for Older Children (PAQ-C) and Adolescents (PAQ-A) Manual. Saskatchewan Uo. 2004.
39. Kruger R, Monyeki MA, Schutte AE, et al. The Exercise, Arterial Modulation and Nutrition in Youth South Africa Study (ExAMIN Youth SA). *Front Pediatr* 2020;8:212.
40. WHO. Global Health Survey (GHS)_Core Modules. 2013
41. Shim JS, Oh K, Kim HC. Dietary assessment methods in epidemiologic studies. *Epidemiol Health* 2014;36:e2014009.
42. The European KIDSCREEN Group. The KIDSCREEN-10 Index: Health-Related Quality of Life Questionnaire for Children and Adolescents aged from 8 to 18 years 2011; Retrieved 13 September 2020; https://www.kidscreen.org/english/questionnaires/kidscreen-10-index/.
43. Ravens-Sieberer U, Erhart M, Rajmil L, et al. Reliability, construct and criterion validity of the KIDSCREEN-10 score: a short measure for children and adolescents' well-being and health-related quality of life. *Qual Life Res* 2010;19(10):1487-500.
44. Reis RS, Hino AA, Anez CR. Perceived stress scale: reliability and validity study in Brazil. *J Health Psychol* 2010;15(1):107-14.
45. Zurlo MC, Pes D, Siegrist J. Validity and reliability of the effort-reward imbalance questionnaire in a sample of 673 Italian teachers. *Int Arch Occup Environ Health* 2010;83(6):665-74.
46. Pujol-Cols L. Development and validation of the Spanish Work-Family Conflict Scale (SP-WFCS): Evidence from two independent samples in Argentina. *Current Psychology* 2019
47. Ülbeğî İ, İplîk, E. The Reliability and Validity of the Shirom-Melamed Burnout Measure. *Cag University Journal of Social Sciences* 2017;14(1):19-30.
48. Forero R, Nahidi S, De Costa J, et al. Application of four-dimension criteria to assess rigour of qualitative research in emergency medicine. *BMC Health Serv Res* 2018;18(1):120.
49. Hsieh HF, Shannon SE. Three approaches to qualitative content analysis. *Qual Health Res* 2005;15(9):1277-88.
50. Glasgow REV, T. M.; Boles S. M. Evaluating the public health impact of health promotion interventions the RE-AIM framework. *Am J Public Health* 1999;89(9):1322-7.
51. Craigie AM, Lake AA, Kelly SA, Adamson AJ, Mathers JC. Tracking of obesity-related behaviours from childhood to adulthood: A systematic review. *Maturitas* 2011;70(3):266-84.
52. WHO. Responding to non-communicable diseases during and beyond the Covid-19 pandemic. 2020
53. Min Y, Perucci F. Impact of COVID-19 on SDG progress: a statistical perspective: United Nations, 2020:5.
54. Spaull N, Van der Berg S. Counting the cost: COVID-19 school closures in South Africa and its impact on children. *South African Journal of Childhood Education* 2020;10(1)
55. Gaglio B. The RE_AIM Framework: A Systematic Review of use over time. 2013;103
56. Lew MS, L'Allemand D, Meli D, et al. Evaluating a childhood obesity program with the Reach, Effectiveness, Adoption, Implementation, Maintenance (RE-AIM) framework. *Prev Med Rep* 2019;13:321-26.
57. Naylor PJ, Nettlefold L, Race D, et al. Implementation of school based physical activity interventions: a systematic review. *Prev Med* 2015;72:95-115.
58. Singh A, Bassi S, Nazar GP, et al. Impact of school policies on non-communicable disease risk factors - a systematic review. *BMC Public Health* 2017;17(1):292.
59. World Health Organization. Growth reference data for 5-19 years: BMI-for-age. 2007; Retrieved 13 September 2020; https://www.who.int/growthref/who2007_bmi_for_age/en/.
60. de Onis M, Onyango AW, Borghi E, et al. Development of a WHO growth reference for school-aged children and adolescents. *Bull World Health Organ* 2007;85(9):660-7.
61. Rosner B, Cook N, Portman R, Daniels S, Falkner B. Determination of blood pressure percentiles in normal-weight children: some methodological issues. *Am J Epidemiol* 2008;167(6):653-66.
62. Flynn JT, Kaelber DC, Baker-Smith CM, et al. Clinical Practice Guideline for Screening and Management of High Blood Pressure in Children and Adolescents. *Pediatrics* 2017;140(3):e20171904.
63. American Diabetes A. 2. Classification and Diagnosis of Diabetes: Standards of Medical Care in Diabetes-2020. *Diabetes Care* 2020;43(Suppl 1):S14-S31.
64. National Cholesterol Education Program (NCEP): Highlights of the Report of the Expert Panel on Blood Cholesterol Levels in Children and Adolescents. *Pediatrics* 1992;89(3):495.
65. Expert Panel on Integrated Guidelines for Cardiovascular H, Risk Reduction in C, Adolescents, National Heart L, Blood I. Expert panel on integrated guidelines for cardiovascular health and risk reduction in children and adolescents: summary report. *Pediatrics* 2011;128 Suppl 5:S213-56.

For peer review only

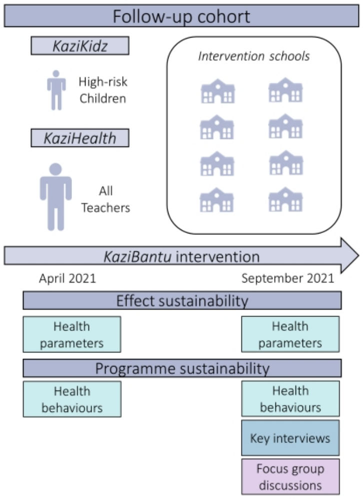

Overview of the follow-up study design to assess the sustainability and effectiveness of KaziKidz and KaziHealth.

Sustainability of a school-based health intervention for prevention of non-communicable diseases in marginalised communities: protocol for a mixed methods cohort study

Arnaiz, Adams et al.

Supplementary Online Material

Information on COVID-19-Related School Lockdown and Closure in South Africa and the Port Elizabeth Region

On 15 March 2020, the President of South Africa, Cyril Ramaphosa, declared a national state of disaster in response to the COVID-19 global pandemic. Ramaphosa announced measures such as immediate travel restrictions and the closure of all schools from 18 March. On 23 March, a national lockdown was announced lasting for 21 days, starting on 27 March. The initial three-week period was eventually extended, with different levels of lockdown regulating the various kinds of restrictions placed people's movement and activity. South Africa developed a 5-tier "Risk Adjusted Strategy Lockdown" categorization [i.e., level 1 (least severe) to level 5 (total lockdown)]. On 01 May 2020, Ramaphosa announced that a gradual and phased easing of lockdown restrictions would be implemented, lowering the national alert level to 4. From 01 June, the national restrictions were lowered to level 3. The restrictions were further lowered to alert level 2 on 17 August. From 21 September, restrictions were lowered again to alert level 1. In December, the country experienced a second wave of COVID-19 infections, resulting in a lockdown level tightening from an adjusted level 1 to an adjusted level 3, starting on 29 December. However, on 01 March 2021, the lockdown level was lowered again from an adjusted level 3 to an adjusted level 1.

The COVID-19 pandemic has affected education systems globally, leading to the near complete closure of some schools. South African schools were closed on 18 March 2020, resuming early June 2020. To compensate for the loss in educational teaching time, the mid-year school holidays were shortened by one week. However, for 10 weeks, children were not permitted to go to school. Although a small proportion of schools could quickly adapt and switch to online learning, educational activity came to a complete stop for most schools and no learning and teaching could take place, especially for quintile three categorization schools and below (i.e., no fee-paying schools), such as those involved in this study. Accordingly, the deep inequalities within the South African schooling system became evident. After several delays, schools officially reopened on 08 June 2020, with a planned staggered return of grades. Learners who returned to school first were those at exit points of primary and high schools, i.e., grades 7 and 12, respectively. However, on 24 July 2020, with only five grades having returned (three for less than one full month), schools were closed again due to an upsurge in COVID-19 cases and deaths. Although schools remained closed until 24 August 2020, grade 12 learners returned to schools on 03 August 2020. On 11 August 2020, grade 7 learners returned to school, whereas on 24 August

For peer review only - http://bmjopen.bmj.com/site/about/guidelines.xhtml

2020, grades R – 4, grade 6, and grades 9 – 11 returned to school. Lastly, grades 5 and 8 returned to school on 31 August 2020.

The most significant educational time loss came from the timetabling model that schools were forced to adopt to meet the social distancing requirements, in addition with the staggered return to school system to further limit the spread of COVID-19. The Department of Basic Education stated that each school must comply with the 1.5m social distancing requirements, as well as all schools must operate at 50%, or less, of their capacity at any given time. Schools were given the liberty to decide how this would be implemented and many schools had varied approaches, incorporating what the schools referred to as a block teaching system. The following were some examples of how the implementation/grouping of the 50% capacity occurred:

- Each class was split in half and would alternate the days learners attend school.
- The entire grade was split in half and would alternate the days each class would attend school.
- Junior and senior grades were split, whereby the junior and senior grades would alternate the days attending school, i.e., all grades 4 and 5 on one day and all grades 6 and 7 the next day.
- A ratio of 3:2 days out of 5 school days was incorporated, whereby one group of learners attended school for the first three days of the week, and then the last 2 days of the following week, and vice versa for the second group.

This 50% capacity system is currently still applicable and being implemented at all schools, particularly those schools involved in this study. Schools were also advised that they should not exclude any of the subjects from the curriculum, but that they should adapt the timetable how they deem it necessary by prioritizing the more heavily weighted academic subjects, such as English, Afrikaans, Mathematics and Science. Consequently, the allocation of certain subjects would have been affected. Furthermore, given that physical education is currently not a stand-alone subject and forms part of the Life Orientation/Life Skills subject, the likelihood of physical education receiving limited attention is high. Although some schools may have attempted to include the Life Orientation/Life Skills subject in the timetable, our assumption is that it may not have been prioritized and may have been neglected by many schools throughout the COVID-19 pandemic. Hence, we believe that the implementation of the *KaziKidz* material would also not necessarily have taken place as originally planned; and requires further exploration.

Although the initial objective of the study was to examine the long-term effectiveness and sustainability of both the *KaziKidz* and *KaziHealth* programs in promoting long lasting, positive lifestyle changes among children and teachers, the objective has now been adapted to evaluate sustainability concerning the continuation of the program implementation, without the assistance from the research team, amidst the COVID-19 pandemic. Therefore, the intervention itself has not been adapted, as the study aims to evaluate its sustainability, whilst embracing the COVID-19 reality.

For peer review only

Lockdown level	Description	Duration
**Level 5**	**Total lockdown**: Drastic measures to contain spread of COVID-19 virus and save lives	26 March 2020 – 30 April 2020
**Level 4**	Extreme precautions to limit community transmission and outbreaks, while allowing some activity to resume	01 May 2020 – 31 May 2020
**Level 3**	Restrictions on many activities, including at workplaces and socially, to address a high risk of transmission	01 June 2020 – 17 August 2020
**Level 2**	Physical distancing and restrictions on leisure and social activities to prevent the resurgence of the virus	18 August 2020 – 20 September 2020
**Level 1**	Most normal activity can resume, with precautions and guidelines followed at all times. Population prepared for an increase in alert levels, if necessary	21 September 2020 – 28 December 2020
**Level 3**	Restrictions on many activities, including at workplaces and socially, to address a high risk of transmission	29 December 2020 – 28 February 2021
**Level 1**	Most normal activity can resume, with precautions and guidelines followed at all times. Population prepared for an increase in alert levels, if necessary	01 March 2021 – Present (May 2021)

**2020 Amended School Calendar**		
**Term**	**Duration**	**Number of school days**
**1**	**15 January – 18 March 2020**	**46**
**2**	**08 June – 24 July 2020**	**34**
**3**	**24 August – 23 October 2020**	**44**
	• 03 – 07 August Grade 12 learners returned	
	• 11 – 14 August: Grade 7 learners returned	
	• 17 – 21 August: Grades 7 and 12 learners returned	
	• 24 August: Grades R – 4, Grade 6 and Grades 9 – 11 learners returned	
	• 31 August: Grades 5 and 8 learners returned	
**4**	**02 November – 15 December 2020**	**32**

**2021 Amended School Calendar**		
**Term**	**Duration**	**Number of school days**
**1**	**15 February – 23 April 2021**	**47**
**2**	**03 May – 19 July 2021**	**49**
**3**	**26 July – 01 October 2021**	**48**
**4**	**11 October –15 December 2021**	**48**

For peer review only - http://bmjopen.bmj.com/site/about/guidelines.xhtml

STROBE Statement—Checklist of items that should be included in reports of ***cohort studies***

	Item No	Recommendation	Page No
**Title and abstract**	1	(*a*) Indicate the study's design with a commonly used term in the title or the abstract	1
		(*b*) Provide in the abstract an informative and balanced summary of what was done and what was found	2-3
**Introduction**			
Background/rationale	2	Explain the scientific background and rationale for the investigation being reported	3-6
Objectives	3	State specific objectives, including any prespecified hypotheses	6
**Methods**			
Study design	4	Present key elements of study design early in the paper	8-9
Setting	5	Describe the setting, locations, and relevant dates, including periods of recruitment, exposure, follow-up, and data collection	6, 8
Participants	6	(*a*) Give the eligibility criteria, and the sources and methods of selection of participants. Describe methods of follow-up	6-7
		(*b*) For matched studies, give matching criteria and number of exposed and unexposed	n/a
Variables	7	Clearly define all outcomes, exposures, predictors, potential confounders, and effect modifiers. Give diagnostic criteria, if applicable	6-7
Data sources/ measurement	8*	For each variable of interest, give sources of data and details of methods of assessment (measurement). Describe comparability of assessment methods if there is more than one group	9-11
Bias	9	Describe any efforts to address potential sources of bias	13
Study size	10	Explain how the study size was arrived at	7
Quantitative variables	11	Explain how quantitative variables were handled in the analyses. If applicable, describe which groupings were chosen and why	11-13
Statistical methods	12	(*a*) Describe all statistical methods, including those used to control for confounding	11-13
		(*b*) Describe any methods used to examine subgroups and interactions	11-13
		(*c*) Explain how missing data were addressed	13
		(*d*) If applicable, explain how loss to follow-up was addressed	13
		(*e*) Describe any sensitivity analyses	n/a
**Results**			
Participants	13*	(a) Report numbers of individuals at each stage of study—eg numbers potentially eligible, examined for eligibility, confirmed eligible, included in the study, completing follow-up, and analysed	n/a
		(b) Give reasons for non-participation at each stage	n/a
		(c) Consider use of a flow diagram	n/a
Descriptive data	14*	(a) Give characteristics of study participants (eg demographic, clinical, social) and information on exposures and potential confounders	n/a
		(b) Indicate number of participants with missing data for each variable of interest	n/a
		(c) Summarise follow-up time (eg, average and total amount)	n/a
Outcome data	15*	Report numbers of outcome events or summary measures over time	n/a

For peer review only

For peer review only - http://bmjopen.bmj.com/site/about/guidelines.xhtml

Main results	16	(*a*) Give unadjusted estimates and, if applicable, confounder-adjusted estimates and their precision (eg, 95% confidence interval). Make clear which confounders were adjusted for and why they were included	n/a
		(*b*) Report category boundaries when continuous variables were categorized	n/a
		(*c*) If relevant, consider translating estimates of relative risk into absolute risk for a meaningful time period	n/a
Other analyses	17	Report other analyses done—eg analyses of subgroups and interactions, and sensitivity analyses	n/a
**Discussion**			
Key results	18	Summarise key results with reference to study objectives	n/a
Limitations	19	Discuss limitations of the study, taking into account sources of potential bias or imprecision. Discuss both direction and magnitude of any potential bias	3, 14
Interpretation	20	Give a cautious overall interpretation of results considering objectives, limitations, multiplicity of analyses, results from similar studies, and other relevant evidence	16
Generalisability	21	Discuss the generalisability (external validity) of the study results	6, 16
**Other information**			
Funding	22	Give the source of funding and the role of the funders for the present study and, if applicable, for the original study on which the present article is based	19

*Give information separately for exposed and unexposed groups.

Note: An Explanation and Elaboration article discusses each checklist item and gives methodological background and published examples of transparent reporting. The STROBE checklist is best used in conjunction with this article (freely available on the Web sites of PLoS Medicine at http://www.plosmedicine.org/, Annals of Internal Medicine at http://www.annals.org/, and Epidemiology at http://www.epidem.com/). Information on the STROBE Initiative is available at http://www.strobe-statement.org.

For peer review only

BMJ Open

Sustainability of a school-based health intervention for prevention of non-communicable diseases in marginalised communities: protocol for a mixed methods cohort study

Journal:	*BMJ Open*
Manuscript ID	bmjopen-2020-047296.R2
Article Type:	Original research
Date Submitted by the Author:	22-Aug-2021
Complete List of Authors:	Arnaiz, Patricia; University of Basel, Department of Sport, Exercise and Health<br>Adams, Larissa; Nelson Mandela Metropolitan University, Department of Human Movement Science<br>Müller, Ivan; University of Basel, Department of Sport, Exercise and Health<br>Gerber, Markus; University of Basel, Department of Sport, Exercise and Health<br>Walter, Cheryl; Nelson Mandela Metropolitan University, Department of Human Movement Science<br>du Randt, Rosa; Nelson Mandela Metropolitan University, Department of Human Movement Science<br>Steinmann, Peter ; Swiss Tropical and Public Health Institute; University of Basel<br>Bergman, Manfred; University of Basel, Department of Social Sciences<br>Seelig, Harald; University of Basel, Department of Sport, Exercise and Health<br>van Greunen, Darelle; Nelson Mandela Metropolitan University, Centre for Community Technologies<br>Utzinger, Jürg; Swiss Tropical and Public Health Institute; University of Basel<br>Pühse, Uwe ; University of Basel, Department of Sport, Exercise and Health
**Primary Subject Heading**:	Public health
Secondary Subject Heading:	Sports and exercise medicine, Evidence based practice, Public health
Keywords:	PREVENTIVE MEDICINE, Community child health < PAEDIATRICS, SPORTS MEDICINE, PUBLIC HEALTH

SCHOLARONE™ Manuscripts

I, the Submitting Author has the right to grant and does grant on behalf of all authors of the Work (as defined in the below author licence), an exclusive licence and/or a non-exclusive licence for contributions from authors who are: i) UK Crown employees; ii) where BMJ has agreed a CC-BY licence shall apply, and/or iii) in accordance with the terms applicable for US Federal Government officers or employees acting as part of their official duties; on a worldwide, perpetual, irrevocable, royalty-free basis to BMJ Publishing Group Ltd ("BMJ") its licensees and where the relevant Journal is co-owned by BMJ to the co-owners of the Journal, to publish the Work in this journal and any other BMJ products and to exploit all rights, as set out in our licence.

The Submitting Author accepts and understands that any supply made under these terms is made by BMJ to the Submitting Author unless you are acting as an employee on behalf of your employer or a postgraduate student of an affiliated institution which is paying any applicable article publishing charge ("APC") for Open Access articles. Where the Submitting Author wishes to make the Work available on an Open Access basis (and intends to pay the relevant APC), the terms of reuse of such Open Access shall be governed by a Creative Commons licence – details of these licences and which Creative Commons licence will apply to this Work are set out in our licence referred to above.

Other than as permitted in any relevant BMJ Author's Self Archiving Policies, I confirm this Work has not been accepted for publication elsewhere, is not being considered for publication elsewhere and does not duplicate material already published. I confirm all authors consent to publication of this Work and authorise the granting of this licence.

For peer review only

Sustainability of a school-based health intervention for prevention of non-communicable diseases in marginalised communities: protocol for a mixed methods cohort study

Patricia Arnaiz[1*] (PhD candidate, patricia.arnaiz@unibas.ch, https://orcid.org/0000-0001-5626-3510), Larissa Adams[2*] (PhD candidate, larissa.adams@mandela.ac.za, https://orcid.org/0000-0003-1459-1916), Ivan Müller[1] (Research associate, ivan.mueller@unibas.ch, https://orcid.org/0000-0002-6397-9979), Markus Gerber[1] (Associate professor, markus.gerber@unibas.ch, https://orcid.org/0000-0001-6140-8948), Cheryl Walter[2] (Head of Department, cheryl.walter@mandela.ac.za), Rosa du Randt[2] (Emeritus professor, rosa.durandt@mandela.ac.za), Peter Steinmann[3,4] (Project leader, peter.steinmann@swisstph.ch, https://orcid.org/0000-0003-4800-3019), Manfred Max Bergman[5] (Professor, max.bergman@unibas.ch, https://orcid.org/0000-0002-4059-5833), Harald Seelig[1] (Statistician, harald.seelig@unibas.ch, https://orcid.org/0000-0002-2178-4807), Darelle van Greunen[6] (Director of Centre, darelle.vangreunen@mandela.ac.za, https://orcid.org/0000-0002-0761-713X), Jürg Utzinger[3,4] (Director of Institute, juerg.utzinger@swisstph.ch), Uwe Pühse[1] (Head of Department, uwe.puehse@unibas.ch, https://orcid.org/0000-0003-1228-316X)

[1]Department of Sport, Exercise and Health, University of Basel, 4052 Basel, Switzerland.

[2]Department of Human Movement Science, Nelson Mandela University, 6031 Gqeberha*, South Africa.

[3]Swiss Tropical and Public Health Institute, 4002 Basel, Switzerland.

[4]University of Basel, 4001 Basel, Switzerland.

[5]Department of Social Sciences, University of Basel, 4051 Basel, Switzerland.
[6]Centre for Community Technologies, Nelson Mandela University, 6031 Gqeberha*, South Africa.
* Formerly known as Port Elizabeth.

Joint first authorship
* PA and LA contributed equally to this paper.
Correspondence to
Patricia Arnaiz patricia.arnaiz@unibas.ch Department of Sport, Exercise and Health, University of Basel, Villa Burckhardt, Gellertstrasse 156, 4052 Basel, Switzerland

Word count 4,212.
Number of tables and figures
Tables 1 and 2; Figure 1.

ABSTRACT

Introduction The prevalence of chronic, lifestyle-related diseases is increasing among adults and children from low- and middle-income countries. Despite the effectiveness of community-based interventions to address this situation, the benefits thereof may disappear in the long-term, due to a lack of maintenance, especially among disadvantaged and high-risk populations. The *KaziBantu* randomized controlled trial conducted in 2019 consisted of two school-based health interventions, *KaziKidz* and *KaziHealth*. The present study will evaluate the long-term effectiveness and sustainability of these interventions in promoting positive lifestyle changes among children and educators in disadvantaged schools in Nelson Mandela Bay, South Africa, in the context of the COVID-19 pandemic.

Methods and analysis This study has an observational, longitudinal, mixed methods design. It will follow up educators and children from the *KaziBantu* study. All 160 educators enrolled in *KaziHealth* will be invited to participate, while the study will focus on 361 *KaziKidz* children (aged 10 to 16 years) identified as having an increased risk for non-communicable diseases. Data collection will take place 1.5 and 2 years post-intervention and includes quantitative and qualitative methods, such as anthropometric measurements, clinical assessments, questionnaires, interviews, and

For peer review only

focus group discussions. Analyses will encompass: prevalence of health parameters; descriptive frequencies of self-reported health behaviours and quality of life; the longitudinal association of these; extent of implementation; personal experiences with the programs; and an impact analysis based on the RE-AIM framework.

Discussion In settings where resources are scarce, sustainable and effective prevention programs are needed. The purpose of this protocol is to outline the design of a study to evaluate *KaziKidz* and *KaziHealth* under real-world conditions in terms of effectiveness, being long-lasting, and becoming institutionalized. We hypothesize that a mixed methods approach will increase understanding of the interventions' capacity to lead to sustainable favourable health outcomes amid challenging environments, thereby generating evidence for policy.

Trial registration number ISRCTN15648510.

STRENGTHS AND LIMITATIONS OF THIS STUDY

- The cohort of children was purposely and objectively identified based on their high-risk profile; the follow-up of personal health outcomes is of additional value.
- The mixed-methods approach, strengthened by the involvement of the community and key stakeholders, will enable a comprehensive evaluation of the programs.
- Internationally recognised measurement methods for quantitative, as well as qualitative, were selected and adapted to the study setting.
- The COVID-19 pandemic might influence schools' decision to discontinue or adapt the post-*KaziBantu* intervention and the research team will assess, but not influence, its implementation.
- The study will yield context-specific results that will be closely linked to COVID-19 measures and practices adopted by the schools.

INTRODUCTION

Socioeconomic, environmental, and cultural factors exert a significant influence on

For peer review only

children's health and well-being.[1] In impoverished communities in low- and middle-income countries (LMICs), children are especially vulnerable to illness and lags in age-appropriate development, negatively affecting their life prospects.[2-4] This precarious situation has been aggravated by the COVID-19 pandemic, which has compromised millions of South African children's education, mental health and nutrition.[5] Furthermore, the disease profile of urban populations from LMICs is approaching that of high-income countries, as the proportion of deaths attributable to chronic, lifestyle-related diseases increases.[6-8] Of particular concern are changing dietary habits and sedentary lifestyle patterns,[9,10] both known to lead to metabolic and physiological changes, such as obesity and hypertension, also in young children.[11,12] Children presenting with cardiometabolic risk factors are at particularly high risk because of their predisposition to developing non-communicable diseases (NCDs) later in life.[13,14] Thus, a reduction of undesired behaviours could potentially prevent the majority of chronic diseases, indicating a great need for prevention programs and awareness campaigns.[15,16]

Global recommendations for physical activity (PA) stipulate a daily minimum of 60 minutes of moderate-to-vigorous PA for children between 5 and 17 years old. Walter and colleagues showed that the levels of in-school PA among children from marginalized communities in South Africa do not meet the minimal requirements,[17] which was later confirmed by *'The Healthy Active Kids South Africa Report Card'* (2018).[18] Therefore, there is an urgent need to promote PA amongst at-risk communities in South Africa. Several school-based lifestyle interventions have proved effective in increasing levels of PA and reducing obesity in children from different contexts.[19-21] However, very few school-based and large-scale PA intervention studies have been conducted in South Africa. The first study, *HealthKick*, was set in disadvantaged low-income communities in Cape Town and aimed at promoting healthy eating habits and regular participation in PA in children to reduce the risk of chronic diseases.[22] Study results were not completely satisfactory and highlighted that educators play a key role in implementing lifestyle interventions in schools.[23,24] Some studies have reported beneficial effects of integrated community-based interventions involving the participation of educators and children.[25-27]

For peer review only

The *'Disease, Activity and Schoolchildren's Health'* (*DASH*) study documented the poor health status and double burden from communicable and non-communicable diseases of children in disadvantaged communities in Gqeberha, formerly known as Port Elizabeth, South Africa.[28] It also revealed the potential for improvement through PA and health literacy interventions, following the principles that PA is key for the promotion of health and well-being among school children, and that learning this at a young age will have long-lasting effects throughout life. Moreover, it emphasized the role of educators as influential and willing advocates.[29] Building on these results and experience, *KaziBantu*, a comprehensive school-based lifestyle intervention program aiming at promoting health literacy in both children and educators of primary schools from disadvantaged communities in Nelson Mandela Bay (NMB) was developed.[30] *KaziBantu*, which is a composite Swahili and isiXhosa phrase meaning «Active People», was designed as a dual approach consisting of two school-based health promotion interventions, *KaziKidz* for children and *KaziHealth* for educators, and was implemented as a cluster randomized controlled trial (RCT) between January and October 2019. Preliminary results show that more than half of the assessed children (544/975) presented at least one risk factor for NCDs. Educators responded positively to the intervention as they felt the program was also focused on their health and well-being (individualised results were communicated to the study participants) and took cognizance of the difficult work environment they faced daily.

Furthermore, evidence suggests that general and work-related stress increases the risk of mental ill-health (burnout, anxiety, impaired quality of sleep), as well as physical disease (coronary heart disease and musculoskeletal disease). These factors seem to contribute to an increased risk of premature death.[31] This risk was confirmed in a large representative sample of South African educators (N=21,307) working in public schools. High stress levels, lack of job satisfaction, and different stress-related physical illnesses (hypertension, heart disease, stomach ulcers, mental distress, tobacco, and alcohol misuse) were reported.[32]

Although evidence exists on the effectiveness of community-based lifestyle interventions in low- and middle-income countries,[33] studies have also shown that positive effects tend to disappear in the long-term,[34] sometimes because programs fail to be maintained.[35,36] In addition, there is still a scarcity of evidence regarding

For peer review only

the feasibility of educational programs in at-risk populations in disadvantaged school settings. We hypothesize that a comprehensive approach focusing specifically on high-risk children and their educators might increase implementation feasibility and ultimately lead to sustainable favourable health outcomes in both groups.

Objectives

The goal of this follow-up study is to determine the long-term post-RCT effectiveness, adoption and continuity of both *KaziKidz* and *KaziHealth* school-based health interventions in disadvantaged primary schools in NMB, South Africa, under real-world conditions and amid the COVID-19 pandemic. To achieve this goal, the following objectives are set:

i. To assess the effectiveness of the interventions in promoting long-lasting, positive lifestyle changes among children and educators, thereby reducing risk factors for NCDs;

ii. To evaluate the post-intervention sustainability concerning the continuation of the program implementation in the schools as well as the institutionalization within the Eastern Cape Department of Education (ECDoE) in South Africa; and

iii. To provide recommendations towards guidelines for a health promotion framework, as well as its adaption to new contexts that may be translated into policy and practice.

METHODS

Research setting

The present study is conducted in the eight schools originally selected for the *KaziBantu* RCT[30]. These are quintile three primary schools in the townships and Northern areas of NMB, in the Eastern Cape province of South Africa. The quintiles of South African schools are determined through the national poverty table and are ranged on a scale from 1 (poorest) to 5 (least poor). Quintile three schools represent no-fee paying schools and their communities are adversely affected by poverty and high unemployment rates.

For peer review only

Study population

Originally, in the *KaziBantu* project, both *KaziKidz* and *KaziHealth* interventions were conducted in four schools, whereas the other four participating schools were used as controls.[30] In total, 975 children from 4th to 6th grade (aged 8 to 16 years) were enrolled in the study; 482 children were allocated to the intervention and 493 to the control arm. At the same time, 160 educators were enrolled among all participating schools; of those, 85 took part in *KaziHealth,* whereas 75 served as waiting-list controls.

This study will follow up children and educators from the *KaziBantu* trial. While all educators who took part in *KaziHealth* have been invited to participate in this follow-up phase, a cohort of children from the *KaziKidz* intervention, who have been identified as having an increased risk for NCDs, have been included in this study. We have defined increased risk as a child presenting at least one of the following cardiometabolic risk factors: [i] overweight or obesity, [ii] elevated blood pressure or hypertension, [iii] pre- or diabetes, [iv] borderline or dyslipidaemia; according to age-appropriate, standard classifications (Table 1) and based on *KaziBantu* baseline data from July 2019. Of the 975 children who participated in *KaziKidz*, 544 were classified as «high risk» according to the above-mentioned definition. Since then, 183 children have graduated from primary school resulting in 361 eligible learners. Of those, 240 have consented to participate and have been enrolled in this follow-up study at the time this protocol was published. Of the 160 educators enrolled in the *KaziHealth* program in 2019, 105 have consented to participate and have been enrolled in the present study so far.

Intervention

The *KaziKidz* intervention consists of a tailored, ready-made teaching material toolkit for grades 1 to 7 and addresses children's risk factors for NCDs, health behaviours, and psychosocial health. On the other hand, *KaziHealth* is designed as a workplace intervention targeting educators' health and well-being through health risk assessments and personalized lifestyle coaching. The interventions have been described in detail elsewhere[30] and all teaching materials and other project resources are freely available online.[37]

For peer review only

After conclusion of the *KaziBantu* RCT in 2019, all eight schools were given the freedom to conduct *KaziKidz* and *KaziHealth*. Thus, all children from grades 1 to 7 may have participated in *KaziKidz*, regardless of health condition. However, the implementation of *KaziKidz* and *KaziHealth* has proceeded independently and without support from the project team in all schools, under so-called «real-world» conditions. Therefore, the delivery of the programs might have been only partially implemented. For instance, some schools might have needed to adapt *KaziKidz* to comply with COVID-19-related requirements, while other schools might have not been able to, or decided not to, continue with the intervention at all.

Study design

This study represents an observational follow-up of the *KaziBantu* RCT and has been designed as a longitudinal study to investigate the long-term effects of both lifestyle interventions, *KaziKidz* and *KaziHealth*, on health parameters and behaviours of a cohort of high-risk children, as well as all educators originally included in the RCT. The overall effectiveness and sustainability of the programs will be assessed using a mixed methods approach, including quantitative and qualitative data collection methods and analyses (Figure 1). Recruitment has started in March 2021. Assessments of health outcomes will be performed both for children and educators at two time-points: April 2021 and September 2021, i.e. 1.5 and 2 years post-RCT, to evaluate the maintenance of healthy, active lifestyles and its effect on an individual level. As part of the post-intervention evaluation, the extent of *KaziKidz*'s implementation in the schools will be obtained from school representatives at both timepoints to be able to correlate the observed outcomes with continued participation in the original intervention. A qualitative analysis of the school headmasters' experiences with *KaziKidz,* as well as of its incorporation into the schools' curriculum with officials from the ECDoE, will take place two years post-RCT, in October 2021. Additionally, qualitative analyses will also be conducted with school educators on their experiences with, and incorporation of, *KaziHealth* into their lifestyles two years post-RCT, in October 2021. Further information on the adoption and acceptance of *KaziKidz* by educators and parents, and of *KaziHealth* by school headmasters' and ECDoE officials will be obtained during October 2021.

For peer review only

This post-intervention sustainability assessment does not involve the evaluation of program delivery adequacy. Similarly, the research team will not influence the effect that «real-world» conditions might have on whether, or how, the interventions are delivered in the schools. Since the studied cohort does not involve the follow-up of a control group, participants from schools refusing the implementation would serve as comparison groups for potential health and behavioural effects.

Data collection

Primary outcomes include anthropometric and clinical examinations, self-reported and objectively measured physical activity, self-reported diet, and self-reported psychosocial health, while secondary outcomes include age, sex, socio-economic status (SES), and school. Standard procedures to assess the interventions' health effects on markers for NCD risk factors will be conducted by well-trained professional staff (e.g. biokineticists), who will adhere to standardized, quality-controlled protocols established during previous work of the research group in the area.

Anthropometric measurements: Body weight will be measured on a digital weighing scale and body height against a stadiometer with back erect and shoulders relaxed. Body mass index (BMI) will be calculated as weight (kg)/height $(m)^2$, and additionally, BMI-for-age stratified by sex will be determined for children according to WHO growth charts. Waist circumference will be measured with a steel tape between the rib cage and the iliac crest and hip circumference at the maximal circumference of the buttocks; waist-to-hip ratio will be calculated as waist circumference/hip circumference. Body fat percentage will be assessed via bioelectrical impedance analysis (BIA) with a wireless body composition monitor (Tanita MC-580; Tanita Corp., Tokyo, Japan) in participants at least 3 hours after food consumption.

Clinical examinations: Blood pressure will be measured three times after the participant has been seating for 5 min. The Omron® digital blood pressure monitor and appropriate cuffs sized 17 to 22 cm for children and 22 to 32 or 32 to 42 cm for adults will be used for this purpose and will be wrapped around the left arm with the bottom of the cuff placed about 4 cm above the elbow and the palm facing up. The blood pressure will be estimated as the average value of the second and third measurement. A full blood lipid profile will assess total cholesterol, low-density

For peer review only

lipoprotein cholesterol (LDL-C), high-density lipoprotein cholesterol (HDL-C), triglycerides, non-HDL cholesterol (non-HDL), and cholesterol to high-density lipoprotein ratio (C-HDL ratio) with the Alere Afinion AS 100 Analyzer® point-of-care testing instrument. Glycated haemoglobin (HbA1c) will be measured from the second of two blood drops extracted from the fingertip previously cleaned with alcohol and pricked with a safety lancet, using the Alere Afinion AS 100 Analyzer®. Haemoglobin concentration will be assessed for educators with the HemoCue Hb 301 system.

Objective measurement of physical behaviours: PA will be measured for each participant using an accelerometer (ActiGraph wGT3X-BT®, Pensacola, Florida, USA) which will be worn around the hip, at all times, for 7 days (5 weekdays and 2 weekend days), except for activities involving water contact.

Questionnaires: Information on PA during school hours and in their free time will be self-reported by children using adjusted questions from the PA Questionnaire for Children (PAQ-C).[38] Dietary behaviours and nutrition will be self-reported by children through a tailored, validated food frequency questionnaire[39,40] and by educators via a 24-hour dietary recall questionnaire.[41] The validated KIDSCREEN-10 questionnaire will be implemented to determine children's physical and psychological well-being.[42,43] Where necessary, questionnaires will be translated into the home language of the children, isiXhosa or Afrikaans, and additionally explained orally by students of the Nelson Mandela University. Self-reported psychosocial health in educators will be assessed through general perceived stress [four-item Perceived Stress Scale (PSS4)],[44] work-related stress [Effort-Reward Imbalance (ERI)],[45] work and family conflict [Work and Family Conflict Scale (WAFCS)],[46] and burnout symptoms (Shirom-Melamed Burnout Measure (SMBM)[47]] questionnaires. Information about educators' work attendance will be obtained from the schools' attendance registers. A self-developed questionnaire will be used to assess the continuity and extent of *KaziKidz*'s implementation in the schools and will be answered by the school representative.

The support for, and integration into the school curriculum of, *KaziKidz* and *KaziHealth* sustainability will be studied by qualitative research, employing credibility, transferability, dependability, confirmability, and authenticity as quality

For peer review only

criteria when employing qualitative methods .[48]

Interviews: Information on the strengths, challenges, and prospects of the *KaziKidz* toolkit implementation in the schools and the Eastern Cape school curricula will be collected via semi-structured interviews. Interviewees will include principals from all participating schools (n=8), as well as representatives from the ECDoE (n=1), Provincial Teacher Development Institute (n=1), and the Nelson Mandela University (n=1). The interview schedule pertains to the perceived strengths, weaknesses, acceptance, and degree of integration of the intervention program into the school curriculum

Focus groups: Information on community feasibility and community acceptance of *KaziKidz* and *KaziHealth* will be gathered during 16 focus group discussion (FGD) sessions of 8 participants each. For *KaziKidz*, 6 focus groups will be organized and will include 24 parents and 24 educators from previous intervention and control schools. The sessions will last approximately 60 minutes. Parents and educators will be part of different groups to focus on the feasibility and acceptance of the program at home and in schools, respectively. For *KaziHealth*, 8 focus groups will include educators from all schools, whereas 2 other sessions will be organized with all 8 schools' principals, on the one hand, and officials from the ECDoE, on the other.

Data management

Throughout the study, personal data will be anonymised. All data will be saved on SWITCHdrive, a Swiss non-commercial cloud data storage service for institutions of higher education. Stored data will be backed up weekly and the principal investigator will supervise the content to ensure data quality. Data collected will be accessible only to authorized personnel (investigators and ethics committee members) and exclusively used for scientific research. Quantitative data will be double-entered and validated in EpiData 3.1 and once cleaned, transferred to STATA for statistical analysis. Interviews and FGDs will be audio-recorded, transcribed, and coded. At the end of the study, data will be anonymized and shared using the publicly accessible data repository, Zenodo.

Data analysis

For peer review only

Anthropometric, clinical indicators and objectively measured PA will be characterized by means and standard deviations, if they are close to the normal distribution, and otherwise by medians and interquartile ranges. Data on self-reported health status, PA, and dietary patterns collected in questionnaires will be treated as categorical data and described by their frequency distributions. The interview and FGD data will be analysed using qualitative content analysis,[49] and each interview and focus group transcript will be cross-coded to locations and thematic fit. To improve inter-coder reliability, two independent coders will be employed. The results of the qualitative analyses will include a taxonomy of the perceived strengths, challenges, and implementation of the intervention program.

Cross-sectional analysis

Mixed linear models: Associations of continuous health and PA outcomes with personal characteristics (e.g. sex, SES) will be assessed post-intervention, i.e. from data collected after the conclusion of the trial. It will be adjusted for the type of school (i.e. intervention vs. control) and potential clustering within schools and classes, respective random intercepts will be used.

Mixed logistic regression: For categorical outcome measures, after suitable dichotomization of polytomous outcomes, associations with the same predictor variables as for the mixed linear models will be analysed post-intervention using the same random intercepts.

Longitudinal analysis

Mixed linear regression: Associations between longitudinal changes in continuous variables (e.g. BMI) and individual characteristics, like sex and SES, will be assessed with random intercepts for schools and classes. Longitudinal changes in these outcomes will also be compared between the previous intervention and control schools after adjustment for personal characteristics. Moreover, changes in health parameters will be regressed against changes in PA and nutritional parameters. All analyses will be conducted, with and without adjustment, for the post-intervention values of the respective outcomes. Also, potential associations between adjusted school-specific means of longitudinal changes in health parameters and the

For peer review only

retrospectively assessed degrees of implementation of intervention measures in the respective schools will be assessed.

Mixed logistic regression: Longitudinal changes in categorical outcomes will be analysed by dichotomizing polytomous outcomes. Analyses will be stratified according to the post-intervention value of the dichotomized outcome, using mixed logistic regression models of the follow-up outcome with the same random effects structure and the same predictor variables, as previously described, for the mixed linear models. Additional analyses will be conducted by treating the post-intervention and the two consequent FU outcomes as repeated measures.

To adjust for biases associated with missing data and/or loss-to-follow-up, inverse probability weighting will be used. Data imputation will be considered as an additional option if the drop-out rate is moderate (i.e. < 20%).

Impact analysis

For the second objective of assessing the overall community impact of the intervention, its acceptability, adoption, implementation, and maintenance will be explored at the individual, community, and organizational level. For this purpose, a systematic analysis will be performed based on the RE-AIM evaluation framework and its proposed dimensions ([i] Reach, [ii] Efficacy, [iii] Adoption, [iv] Implementation, and [v] Maintenance).[50] Each dimension will be analysed separately, using pieces of data collected by the above-mentioned methods and as described in Table 2, and results will be expressed as percentages. Ultimately, the combination of all five dimensions will result in a score that Glasgow and colleagues refer to as «public health impact» or «population-based effect».[50]

Participant and public involvement

Adult participants were involved in the conception of the research question by identifying their pivotal role in both the implementation and maintenance of the intervention. They will collaborate in the methodology via interviews and FGDs, where preliminary results will be presented for their verification. Participants will also contribute to the results by reporting on the burden of the interventions and specific local needs.

For peer review only - http://bmjopen.bmj.com/site/about/guidelines.xhtml

Ethics and dissemination

The proposed research will be carried out according to the international ethical, scientific and clinical practice standards established in the Declaration of Helsinki, gathered in the International Conference on Harmonization (ICH) Guidelines, and encouraged by the WHO. Prior to study conduct, ethical approval was granted by the Nelson Mandela University Research Ethics Committee (ref. no. H20-HEA-HMS-001; 21/07/2020), the Ethics Committee Northwest and Central Switzerland (EKNZ; ref. no. Req-2020-00430; 12/05/2020) and the Eastern Cape Department of Education (07/12/2020). Furthermore, the study has been registered in ISRCTN (reference number ISRCTN15648510; 17/04/2020), a primary registry on the WHO International Clinical Trials Registry Platform (ICTRP). Participants have been informed on the study, alongside their right to voluntary participation and withdrawal at any time without justification. Before taking part in any assessment, as proposed by this study, oral assent has been sought from children and written consent from their guardians, as well as from the participating educators.

Study results will be summarized in manuscripts and submitted for publication to open-access, peer-reviewed scientific journals. Results will also be presented at relevant national and international conferences and workshops.

Methodological limitations

The implementation of *KaziKidz* and *KaziHealth* will not be monitored by the research team and schools were given the freedom to decide whether, and to what extent, they will deliver the health interventions. Therefore, any participating school may decide not to implement the interventions after completion of the *KaziBantu* trial. The extent of *KaziKidz*'s implementation will be assessed via questionnaire with school representatives and information about the reasons for discontinuation will be sought; this information will be considered for the feasibility assessment. Similarly, the status and extent, including reasons for discontinuation, of *KaziHealth*'s implementation will also be explored and evaluated via FGDs with school educators, headmasters' and ECDoE officials. Furthermore, for those cases where a further implementation of the interventions has not taken place, it will be aimed at measuring

For peer review only

the corresponding children and educators at the foreseen follow-up time-points.

DISCUSSION

Globally, a total of 41 million deaths are attributable to non-communicable, chronic conditions, such as diabetes, coronary heart diseases, or cancer every year. Of those deaths, it is estimated that around 80% are preventable because they are associated with poor diets, physical inactivity, alcohol addiction, tobacco use, and polluted environments. However, NCD aetiology is very complex and individuals cannot be held responsible because they live in a system that encourages unhealthy behaviours. Hence, aiming merely to change behaviours might be complicated and ineffective over time. Instead, the focus should be on tailoring interventions aimed at creating environments that promote healthy lifestyles, making the system more beneficial, accessible, and affordable for everyone. This is especially important in the early stages of life, where healthy behaviours can be adopted naturally and maintained throughout life. Furthermore, NCDs are a major threat to individual and national economic development: unhealthy habits acquired during childhood will most likely lead to unhealthy adults who are unable to develop to their full potential.[51]

The COVID-19 pandemic has highlighted the increased risk for severe disease and mortality faced by people living with chronic conditions.[52] At the same time, it has jeopardized the livelihood of millions of families, disproportionately hitting the most vulnerable and taking a special toll on children.[53] By December 2020, South African children lost up to a third of the school year, thus, not being able to complete the entire curriculum and leaving many gaps in children's education. Moreover, socioeconomic inequality is also likely to have further increased, since poorer learners and schools were least able to catch up.[54]

Upon re-opening in both 2020 and 2021, schools in South Africa have been forced to adopt strict measures to further limit the spread of COVID-19. In addition to the staggered return to the school system, the 1.5-meter social distancing requirement has led to schools incorporating what they referred to as a block teaching system, whereby schools operate at 50% or less of their capacity. More information on COVID-19-related school lockdown and closure in South Africa and the Gqeberha region can be found in the supplementary online material. Furthermore, the Eastern

For peer review only

Cape Department of Basic Education advised schools not to exclude any of the subjects from the curriculum, but to prioritize the more heavily weighted academic subjects, such as English, Afrikaans, Mathematics and Science. Physical education is currently not a stand-alone subject in the South African curriculum but forms part of the Life Orientation/Life Skills subject. Consequently, the likelihood of physical education receiving limited attention, and even being neglected, throughout the COVID-19 pandemic is high. Hence, we believe that the implementation of the *KaziKidz* material might have been affected too, which justifies further exploration. The complexity of lifestyle interventions, such as *KaziKidz* and *KaziHealth,* and their successful implementation reside in their multi-dimensional nature, that is, the involvement of individual, environmental and policy factors that interact at multiple social levels (individual, community, and organizational institutional). The RE-AIM framework will be followed to predict the feasibility of both interventions in terms of effectiveness, addressing whole populations, being long-lasting, and becoming institutionalized.[50,55,56] The information obtained from linking the intervention integration level in the schools with the personal adoption of behavioural changes and the achieved health effects both in children and educators, will yield unprecedented results on lifestyle interventions' potential to effectively promote the acquisition and maintenance of healthy lifestyles during childhood and adulthood that will ultimately support overall community health and well-being. Furthermore, we believe that continuing with the assessments indicated in the methodology sections will provide valuable insights into the children's health amidst the COVID-19 pandemic, and facilitate contextually valid recommendations for future interventions, policy and practice.

Finally, due to the multi-factorial nature of NCDs, collaboration among different sectors, such as governments, communities, academia, and the private sector, is necessary. The comprehensive evaluation of a tailored school-based health promotion intervention presented in this paper could provide unique evidence and support the dissemination of the intervention and collaboration between partners from different sectors,[57] ultimately influencing local policy.[58]

TABLES AND FIGURES

For peer review only

Table 1 Classification criteria for cardiometabolic risk in children

Risk factor	Indicators and cut-off points
**Adiposity**[59,60]	**BMI-for-age** (SD)
Overweight	>+1 and ≤+2
Obesity	>+2
**Blood pressure**[61,62]	**SBP and/or DBP** (percentiles and mmHg)
Elevated blood pressure	≥90th and <95th or <90th but ≥120/80
Hypertension stage 1	≥95th and ≤99th or ≥130/80
Hypertension stage 2	>99th or ≥140/90
**Glycaemia**[63]	**HbA1c** (mmol/mol)
Pre-diabetes	>39 and <48
Diabetes	≥48
**Dyslipidaemia**[64,65]	**TC** (mmol/l)
Borderline	≥4.40 and 5.15
Dyslipidaemia	≥5.15

BMI: body mass index; SD: standard deviation; SBP: systolic blood pressure; DBP: diastolic blood pressure; HbA1c: glycated haemoglobin; TC: total cholesterol.

Table 2 Impact analysis evaluation based on the RE-AIM framework.

Dimensions	Social level	Evaluation criteria
**Reach**<br>Proportion of the target population that has participated in the intervention	**Individual**<br>Children<br>Educators	Number and characteristics of children who:<br>➢ Have received at least half the planned lessons, as reported by the educators<br>Number and characteristics of educators who:

For peer review only

		➢ Have received the health risk assessment<br>➢ Have attended at least one face-to-face coaching session
**Efficacy**<br>Success rate of the intervention in reducing risk factors	**Individual**<br>Children<br>Educators	Changes in:<br>➢ Anthropometric measurements<br>➢ Clinical parameters<br>➢ Quality of life
**Adoption**<br>Proportion of settings that has incorporated the intervention	**Organizational**<br>School educators<br>School principals	Number and characteristics of:<br>➢ Schools that implemented the intervention<br>➢ Health-promoting activities per school
	**Community**<br>School educators<br>Parents	Number and characteristics of:<br>➢ Educators who have adopted *KaziHealth* recommendations<br>➢ Parents aware of and supporting *KaziKidz* activities
**Implementation**<br>Extent to which the intervention has been embedded in the schools under naturalistic conditions	**Organizational**<br>School principals	Number of:<br>➢ Grades and classes participating in the intervention<br>➢ Terms per school year in which the intervention is being taught<br>➢ Staff involved in delivering the intervention
**Maintenance**<br>Adherence to and dissemination of the intervention	**Individual**<br>Children<br>Educators	Changes in:<br>➢ Physical activity and dietary behaviour
	**Organizational**<br>Eastern Cape	Number and characteristics of:

For peer review only

	Department of Education Nelson Mandela University	➢ Schools/districts that have the intervention integrated into the curriculum/environment<br>➢ Actions taken towards its permanent integration (policy), e.g. teaching agenda of future educators

Figure 1 Overview of the follow-up study design to assess the sustainability and effectiveness of *KaziKidz* and *KaziHealth*.

Acknowledgements

The research team would like to thank Dr Bruce Damons (Director of Centre for the Community School, Nelson Mandela University, Gqeberha) for acting as an advisor on the project and being a key intermediary between the universities and the schools. We would also like to thank PD Dr Christian Schindler, who provided input for the statistical analysis of the study. This study takes place under the auspices of the UNESCO Chair on *'Physical Activity and Health in Educational Settings*'.

Contributors

PA, LA, and IM conceptualised the study and led the development of the general project. MG, UP, CW, RdR, PS, DvG and JU contributed to the development of the study design and methodology. MB and HS provided statistical input and developed the draft data analysis plan. PA and LA drafted this manuscript, PA elaborated the *KaziKidz*'s part, while LA contributed with the *KaziHealth*'s fraction. All co-authors commented on revised drafts and approved the final version of the manuscript.

Data sharing

Individual de-identified participant data will be shared using the publicly accessible data repository Zenodo. Data will become available at the end of the study for at least 10 years.

For peer review only

Funding

This study is funded by the Swiss National Science Foundation (SNF; Bern, Switzerland; project no. 320030_192651).

Competing interests

All authors have completed the ICMJE uniform disclosure form at www.icmje.org/coi_disclosure.pdf and declare: PA and UP received financial support from the SNF for the submitted work; no financial relationships with any organizations that might have an interest in the submitted work in the previous three years; no other relationships or activities that could appear to have influenced the submitted work.

Provenance and peer review

Not commissioned; externally peer-reviewed.

REFERENCES

1. Blair DM, Stewart-Brown PS, Waterston DT, Crowther DR. Child Public Health, 2010.
2. Steinmann P, Usubalieva J, Imanalieva C, et al. Rapid appraisal of human intestinal helminth infections among schoolchildren in Osh oblast, Kyrgyzstan. *Acta Trop* 2010;116(3):178-84.
3. Victora CG, Adair L, Fall C, et al. Maternal and child undernutrition: consequences for adult health and human capital. *The Lancet* 2008;371(9609):340-57.
4. Dewey KG, Begum K. Long-term consequences of stunting in early life. *Matern Child Nutr* 2011;7 Suppl 3:5-18.
5. Zar HJ, Dawa J, Fischer GB, Castro-Rodriguez JA. Challenges of COVID-19 in children in low- and middle-income countries. *Paediatr Respir Rev* 2020;35:70-74.
6. Collaboration NCDRF. Rising rural body-mass index is the main driver of the global obesity epidemic in adults. *Nature* 2019;569(7755):260-64.
7. Gouda HN, Charlson F, Sorsdahl K, et al. Burden of non-communicable diseases in sub-Saharan Africa, 1990–2017: results from the Global Burden of Disease Study 2017. *The Lancet Global Health* 2019;7(10):e1375-e87.
8. Lozano R, Naghavi M, Foreman K, et al. Global and regional mortality from 235 causes of death for 20 age groups in 1990 and 2010: a systematic analysis for the Global Burden of Disease Study 2010. *The Lancet* 2012;380(9859):2095-128.
9. Lim SS, Vos T, Flaxman AD, et al. A comparative risk assessment of burden of disease and injury attributable to 67 risk factors and risk factor clusters in 21 regions, 1990–2010: a systematic analysis for the Global Burden of Disease Study 2010. *The Lancet* 2012;380(9859):2224-60.
10. Murray CJL, Vos T, Lozano R, et al. Disability-adjusted life years (DALYs) for 291 diseases and injuries in 21 regions, 1990–2010: a systematic analysis for the Global Burden of Disease Study 2010. *The Lancet* 2012;380(9859):2197-223.
11. Steyn NP, McHiza ZJ. Obesity and the nutrition transition in Sub-Saharan Africa. *Ann N Y Acad Sci* 2014;1311:88-101.
12. Moselakgom VK, Staden MV. Physical Activity and Risk Factors of Cardio-metabolic Diseases in South African Children. *Asian Journal of Scientific Research* 2018;11(4):498-505.
13. Berenson GS, Srinivasan SR, Bao W, et al. Association between Multiple Cardiovascular Risk Factors and Atherosclerosis in Children and Young Adults. *New England Journal of Medicine* 1998;338(23):1650-56.
14. Joshi SM, Katre PA, Kumaran K, et al. Tracking of cardiovascular risk factors from childhood to young adulthood — the Pune Children's Study. *International Journal of Cardiology* 2014;175(1):176-78.
15. Yawson AE, Abuosi AA, Badasu DM, et al. Non-communicable diseases among children in Ghana: health and social concerns of parent/caregivers. *Afr Health Sci* 2016;16(2):378-88.
16. WHO. Global Strategy on Diet, Physical Activity and Health. 2004
17. Walter CM. In-school physical activity patterns of primary school learners from disadvantaged schools in South Africa. *African Journal for Physical, Health Education, Recreation and Dance (AJPHERD)* 2011;17(4):780-89.
18. Draper CE. Results from the Healthy Active Kids South Africa 2018 Report Card. *S Afr J Child Health* 2018;13(3):130-36.
19. Puder JJ, Marques-Vidal P, Schindler C, et al. Effect of multidimensional lifestyle intervention on fitness and adiposity in predominantly migrant preschool children (Ballabeina): cluster randomised controlled trial. *BMJ* 2011;343:d6195.
20. Wilson DK, Van Horn ML, Kitzman-Ulrich H, et al. Results of the "Active by Choice Today" (ACT) randomized trial for increasing physical activity in low-income and minority adolescents. *Health Psychol* 2011;30(4):463-71.
21. Lambrinou CP, Androutsos O, Karaglani E, et al. Effective strategies for childhood obesity prevention via school based, family involved interventions: a critical review for the development of the Feel4Diabetes-study school based component. *BMC Endocr Disord* 2020;20(Suppl 2):52.
22. Draper CE, de Villiers A, Lambert EV, et al. HealthKick: a nutrition and physical activity intervention for primary schools in low-income settings. *BMC Public Health* 2010;10(1):398.
23. de Villiers A, Steyn NP, Draper CE, et al. Implementation of the HealthKick intervention in primary schools in low-income settings in the

For peer review only

Western Cape Province, South Africa: a process evaluation. *BMC Public Health* 2015;15:818.
24. Blaine RE, Franckle RL, Ganter C, et al. Using School Staff Members to Implement a Childhood Obesity Prevention Intervention in Low-Income School Districts: the Massachusetts Childhood Obesity Research Demonstration (MA-CORD Project), 2012-2014. *Prev Chronic Dis* 2017;14:E03.
25. Gorely T, Nevill ME, Morris JG, Stensel DJ, Nevill A. Effect of a school-based intervention to promote healthy lifestyles in 7-11 year old children. *Int J Behav Nutr Phys Act* 2009;6:5.
26. Rosario R, Oliveira B, Araujo A, et al. The impact of an intervention taught by trained teachers on childhood overweight. *Int J Environ Res Public Health* 2012;9(4):1355-67.
27. Innerd AL, Azevedo LB, Batterham AM. The effect of a curriculum-based physical activity intervention on accelerometer-assessed physical activity in schoolchildren: A non-randomised mixed methods controlled before-and-after study. *PLoS One* 2019;14(12):e0225997.
28. Gerber M, Müller I, Walter C, et al. Physical activity and dual disease burden among South African primary schoolchildren from disadvantaged neighbourhoods. *Prev Med* 2018;112:104-10.
29. Müller I, Schindler C, Adams L, et al. Effect of a Multidimensional Physical Activity Intervention on Body Mass Index, Skinfolds and Fitness in South African Children: Results from a Cluster-Randomised Controlled Trial. *Int J Environ Res Public Health* 2019;16(2)
30. Müller I, Smith D, Adams L, et al. Effects of a School-Based Health Intervention Program in Marginalized Communities of Port Elizabeth, South Africa (the KaziBantu Study): Protocol for a Randomized Controlled Trial. *JMIR Res Protoc* 2019;8(7):e14097.
31. Kivimäki M, Leino-Arjas P, Luukkonen R, et al. Work stress and risk of cardiovascular mortality: prospective cohort study of industrial employees. *BMJ* 2002;325(7369):857.
32. Peltzer K, Shisana O, Zuma K, Van Wyk B, Zungu-Dirwayi N. Job stress, job satisfaction and stress-related illnesses among South African educators. *Stress and Health* 2009;25(3):247-57.
33. Sarrafzadegan N, Kelishadi R, Esmaillzadeh A, et al. Do lifestyle interventions work in developing countries? Findings from the Isfahan Healthy Heart Program in the Islamic Republic of Iran. *Bull World Health Organ* 2009;87(1):39-50.
34. Gerber M, Fuchs R, Pühse U. Follow-up of a short motivational and volitional exercise-intervention trial with overweight and obese individuals. *Schweizerische Zeitschrift für «Sportmedizin und Sporttraumatologie»* 2010;58(4):108–14.
35. Sarrafzadegan N, Rabiei K, Wong F, et al. The sustainability of interventions of a community-based trial on children and adolescents' healthy lifestyle. *ARYA Atheroscler* 2014;10(2):107-17.
36. Nguyen S, Hacker AL, Henderson M, et al. Physical Activity Programs with Post-Intervention Follow-Up in Children: A Comprehensive Review According to Categories of Intervention. *Int J Environ Res Public Health* 2016;13(7)
37. UNESCO Chair on Physical Activity and Health in Educational Settings. KaziBantu project and teaching materials. 2019; Retrieved 13 September 2020; https://kazibantu.org/.
38. Kowalski KC, Crocker PRE, Donen RM. The Physical Activity Questionnaire for Older Children (PAQ-C) and Adolescents (PAQ-A) Manual. Saskatchewan Uo. 2004.
39. Kruger R, Monyeki MA, Schutte AE, et al. The Exercise, Arterial Modulation and Nutrition in Youth South Africa Study (ExAMIN Youth SA). *Front Pediatr* 2020;8:212.
40. WHO. Global Health Survey (GHS)_Core Modules. 2013
41. Shim JS, Oh K, Kim HC. Dietary assessment methods in epidemiologic studies. *Epidemiol Health* 2014;36:e2014009.
42. The European KIDSCREEN Group. The KIDSCREEN-10 Index: Health-Related Quality of Life Questionnaire for Children and Adolescents aged from 8 to 18 years 2011; Retrieved 13 September 2020; https://www.kidscreen.org/english/questionnaires/kidscreen-10-index/.
43. Ravens-Sieberer U, Erhart M, Rajmil L, et al. Reliability, construct and criterion validity of the KIDSCREEN-10 score: a short measure for children and adolescents' well-being and health-related quality of life. *Qual Life Res* 2010;19(10):1487-500.
44. Reis RS, Hino AA, Anez CR. Perceived stress scale: reliability and validity study in Brazil. *J Health Psychol* 2010;15(1):107-14.
45. Zurlo MC, Pes D, Siegrist J. Validity and reliability of the effort-reward imbalance questionnaire in a sample of 673 Italian teachers. *Int Arch Occup Environ Health* 2010;83(6):665-74.
46. Pujol-Cols L. Development and validation of the Spanish Work-Family Conflict Scale (SP-WFCS): Evidence from two independent samples in Argentina. *Current Psychology* 2019
47. Ülbeğî İ, İplîk, E. The Reliability and Validity of the Shirom-Melamed Burnout Measure. *Cag University Journal of Social Sciences* 2017;14(1):19-30.
48. Forero R, Nahidi S, De Costa J, et al. Application of four-dimension criteria to assess rigour of qualitative research in emergency medicine. *BMC Health Serv Res* 2018;18(1):120.
49. Hsieh HF, Shannon SE. Three approaches to qualitative content analysis. *Qual Health Res* 2005;15(9):1277-88.
50. Glasgow REV, T. M.; Boles S. M. Evaluating the public health impact of health promotion interventions the RE-AIM framework. *Am J Public Health* 1999;89(9):1322-7.
51. Craigie AM, Lake AA, Kelly SA, Adamson AJ, Mathers JC. Tracking of obesity-related behaviours from childhood to adulthood: A systematic review. *Maturitas* 2011;70(3):266-84.
52. WHO. Responding to non-communicable diseases during and beyond the Covid-19 pandemic. 2020
53. Min Y, Perucci F. Impact of COVID-19 on SDG progress: a statistical perspective: United Nations, 2020:5.
54. Spaull N, Van der Berg S. Counting the cost: COVID-19 school closures in South Africa and its impact on children. *South African Journal of Childhood Education* 2020;10(1)
55. Gaglio B. The RE_AIM Framework: A Systematic Review of use over time. 2013;103
56. Lew MS, L'Allemand D, Meli D, et al. Evaluating a childhood obesity program with the Reach, Effectiveness, Adoption, Implementation, Maintenance (RE-AIM) framework. *Prev Med Rep* 2019;13:321-26.
57. Naylor PJ, Nettlefold L, Race D, et al. Implementation of school based physical activity interventions: a systematic review. *Prev Med* 2015;72:95-115.
58. Singh A, Bassi S, Nazar GP, et al. Impact of school policies on non-communicable disease risk factors - a systematic review. *BMC Public Health* 2017;17(1):292.
59. World Health Organization. Growth reference data for 5-19 years: BMI-for-age. 2007; Retrieved 13 September 2020; https://www.who.int/growthref/who2007_bmi_for_age/en/.
60. de Onis M, Onyango AW, Borghi E, et al. Development of a WHO growth reference for school-aged children and adolescents. *Bull World Health Organ* 2007;85(9):660-7.
61. Rosner B, Cook N, Portman R, Daniels S, Falkner B. Determination of blood pressure percentiles in normal-weight children: some methodological issues. *Am J Epidemiol* 2008;167(6):653-66.
62. Flynn JT, Kaelber DC, Baker-Smith CM, et al. Clinical Practice Guideline for Screening and Management of High Blood Pressure in Children and Adolescents. *Pediatrics* 2017;140(3):e20171904.
63. American Diabetes A. 2. Classification and Diagnosis of Diabetes: Standards of Medical Care in Diabetes-2020. *Diabetes Care* 2020;43(Suppl 1):S14-S31.
64. National Cholesterol Education Program (NCEP): Highlights of the Report of the Expert Panel on Blood Cholesterol Levels in Children and Adolescents. *Pediatrics* 1992;89(3):495.
65. Expert Panel on Integrated Guidelines for Cardiovascular H, Risk Reduction in C, Adolescents, National Heart L, Blood I. Expert panel on integrated guidelines for cardiovascular health and risk reduction in children and adolescents: summary report. *Pediatrics* 2011;128 Suppl 5:S213-56.

For peer review only

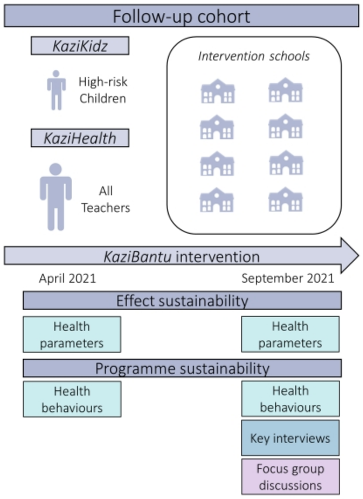

Overview of the follow-up study design to assess the sustainability and effectiveness of KaziKidz and KaziHealth.

Sustainability of a school-based health intervention for prevention of non-communicable diseases in marginalised communities: protocol for a mixed methods cohort study

Arnaiz, Adams et al.

Supplementary Online Material

Information on COVID-19-Related School Lockdown and Closure in South Africa and the Port Elizabeth Region

On 15 March 2020, the President of South Africa, Cyril Ramaphosa, declared a national state of disaster in response to the COVID-19 global pandemic. Ramaphosa announced measures such as immediate travel restrictions and the closure of all schools from 18 March. On 23 March, a national lockdown was announced lasting for 21 days, starting on 27 March. The initial three-week period was eventually extended, with different levels of lockdown regulating the various kinds of restrictions placed people's movement and activity. South Africa developed a 5-tier "Risk Adjusted Strategy Lockdown" categorization [i.e., level 1 (least severe) to level 5 (total lockdown)]. On 01 May 2020, Ramaphosa announced that a gradual and phased easing of lockdown restrictions would be implemented, lowering the national alert level to 4. From 01 June, the national restrictions were lowered to level 3. The restrictions were further lowered to alert level 2 on 17 August. From 21 September, restrictions were lowered again to alert level 1. In December, the country experienced a second wave of COVID-19 infections, resulting in a lockdown level tightening from an adjusted level 1 to an adjusted level 3, starting on 29 December. However, on 01 March 2021, the lockdown level was lowered again from an adjusted level 3 to an adjusted level 1. On 31 May 2021 the country was moved from adjusted level 1 to an adjusted alert level 2, due to a third wave of infections. On 15 June 2021 the country was moved to alert level 3. On 28 June 2021, the country was moved to adjusted level 4, with the Delta variant fast becoming the dominant strain in the country. On 25 July the country was moved to adjusted level 3.

The COVID-19 pandemic has affected education systems globally, leading to the near complete closure of some schools. South African schools were closed on 18 March 2020, resuming early June 2020. To compensate for the loss in educational teaching time, the mid-year school holidays were shortened by one week. However, for 10 weeks, children were not permitted to go to school. Although a small proportion of schools could quickly adapt and switch to online learning, educational activity came to a complete stop for most schools and no learning and teaching could take place, especially for quintile three categorization schools and below (i.e., no fee-paying schools), such as those involved in this study. Accordingly, the deep inequalities within the South African schooling system became evident. After several delays, schools officially reopened on 08 June 2020, with a planned staggered return of grades. Learners who returned to school first were those at exit points of primary and high schools, i.e., grades 7 and 12, respectively. However, on 24 July 2020, with only

For peer review only

For peer review only - http://bmjopen.bmj.com/site/about/guidelines.xhtml

five grades having returned (three for less than one full month), schools were closed again due to an upsurge in COVID-19 cases and deaths. Although schools remained closed until 24 August 2020, grade 12 learners returned to schools on 03 August 2020. On 11 August 2020, grade 7 learners returned to school, whereas on 24 August 2020, grades R – 4, grade 6, and grades 9 – 11 returned to school. Lastly, grades 5 and 8 returned to school on 31 August 2020.

The most significant educational time loss came from the timetabling model that schools were forced to adopt to meet the social distancing requirements, in addition with the staggered return to school system to further limit the spread of COVID-19. The Department of Basic Education stated that each school must comply with the 1.5m social distancing requirements, as well as all schools must operate at 50%, or less, of their capacity at any given time. Schools were given the liberty to decide how this would be implemented and many schools had varied approaches, incorporating what the schools referred to as a block teaching system. The following were some examples of how the implementation/grouping of the 50% capacity occurred:

- Each class was split in half and would alternate the days learners attend school.
- The entire grade was split in half and would alternate the days each class would attend school.
- Junior and senior grades were split, whereby the junior and senior grades would alternate the days attending school, i.e., all grades 4 and 5 on one day and all grades 6 and 7 the next day.
- A ratio of 3:2 days out of 5 school days was incorporated, whereby one group of learners attended school for the first three days of the week, and then the last 2 days of the following week, and vice versa for the second group.

This 50% capacity system is currently still applicable and being implemented at all schools, particularly those schools involved in this study. Schools were also advised that they should not exclude any of the subjects from the curriculum, but that they should adapt the timetable how they deem it necessary by prioritizing the more heavily weighted academic subjects, such as English, Afrikaans, Mathematics and Science. Consequently, the allocation of certain subjects would have been affected. Furthermore, given that physical education is currently not a stand-alone subject and forms part of the Life Orientation/Life Skills subject, the likelihood of physical education receiving limited attention is high. Although some schools may have attempted to include the Life Orientation/Life Skills subject in the timetable, our assumption is that it may not have been prioritized and may have been neglected by many schools throughout the COVID-19 pandemic. Hence, we believe that the implementation of the *KaziKidz* material would also not necessarily have taken place as originally planned; and requires further exploration.

Although the initial objective of the study was to examine the long-term effectiveness and sustainability of both the *KaziKidz* and *KaziHealth* programs in promoting long lasting, positive lifestyle changes among children and teachers, the objective has now been adapted to evaluate sustainability concerning the continuation of the program

For peer review only - http://bmjopen.bmj.com/site/about/guidelines.xhtml

implementation, without the assistance from the research team, amidst the COVID-19 pandemic. Therefore, the intervention itself has not been adapted, as the study aims to evaluate its sustainability, whilst embracing the COVID-19 reality.

Lockdown level	Description	Duration
**Level 5**	**Total lockdown**: Drastic measures to contain spread of COVID-19 virus and save lives	26 March 2020 – 30 April 2020
**Level 4**	Extreme precautions to limit community transmission and outbreaks, while allowing some activity to resume	01 May 2020 – 31 May 2020
**Level 3**	Restrictions on many activities, including at workplaces and socially, to address a high risk of transmission	01 June 2020 – 17 August 2020
**Level 2**	Physical distancing and restrictions on leisure and social activities to prevent the resurgence of the virus	18 August 2020 – 20 September 2020
**Level 1**	Most normal activity can resume, with precautions and guidelines followed at all times. Population prepared for an increase in alert levels, if necessary	21 September 2020 – 28 December 2020
**Level 3**	Restrictions on many activities, including at workplaces and socially, to address a high risk of transmission	29 December 2020 – 28 February 2021
**Level 1**	Most normal activity can resume, with precautions and guidelines followed at all times. Population prepared for an increase in alert levels, if necessary	01 March 2021 – 30 May 2021
**Level 2**	Physical distancing and restrictions on leisure and social activities to prevent the resurgence of the virus	31 May 2021 – 15 June 2021
**Level 3**	Restrictions on many activities, including at workplaces and socially, to address a high risk of transmission	16 June 2021 – 27 June 2021
**Level 4**	Extreme precautions to limit community transmission and outbreaks, while allowing some activity to resume	28 June 2021 – 24 July 2021
**Level 3**	Restrictions on many activities, including at workplaces and socially, to address a high risk of transmission	25 July 2021 – Present (August 2021)

2020 Amended School Calendar		
**Term**	**Duration**	**Number of school days**

For peer review only

1	**15 January – 18 March 2020**	**46**
2	**08 June – 24 July 2020**	**34**
3	**24 August – 23 October 2020**	**44**
	• 03 – 07 August Grade 12 learners returned	
	• 11 – 14 August: Grade 7 learners returned	
	• 17 – 21 August: Grades 7 and 12 learners returned	
	• 24 August: Grades R – 4, Grade 6 and Grades 9 – 11 learners returned	
	• 31 August: Grades 5 and 8 learners returned	
4	**02 November – 15 December 2020**	**32**

**2021 Amended School Calendar**		
**Term**	**Duration**	**Number of school days**
1	**15 February – 23 April 2021**	**47**
2	**03 May – 09 July 2021**	**39**
3	**26 July – 01 October 2021**	**48**
4	**11 October –15 December 2021**	**48**

For peer review only

STROBE Statement—Checklist of items that should be included in reports of ***cohort studies***

	Item No	Recommendation	Page No
**Title and abstract**	1	(*a*) Indicate the study's design with a commonly used term in the title or the abstract	1
		(*b*) Provide in the abstract an informative and balanced summary of what was done and what was found	2-3
**Introduction**			
Background/rationale	2	Explain the scientific background and rationale for the investigation being reported	3-6
Objectives	3	State specific objectives, including any prespecified hypotheses	6
**Methods**			
Study design	4	Present key elements of study design early in the paper	8-9
Setting	5	Describe the setting, locations, and relevant dates, including periods of recruitment, exposure, follow-up, and data collection	6, 8
Participants	6	(*a*) Give the eligibility criteria, and the sources and methods of selection of participants. Describe methods of follow-up	6-7
		(*b*) For matched studies, give matching criteria and number of exposed and unexposed	n/a
Variables	7	Clearly define all outcomes, exposures, predictors, potential confounders, and effect modifiers. Give diagnostic criteria, if applicable	6-7
Data sources/ measurement	8*	For each variable of interest, give sources of data and details of methods of assessment (measurement). Describe comparability of assessment methods if there is more than one group	9-11
Bias	9	Describe any efforts to address potential sources of bias	13
Study size	10	Explain how the study size was arrived at	7
Quantitative variables	11	Explain how quantitative variables were handled in the analyses. If applicable, describe which groupings were chosen and why	11-13
Statistical methods	12	(*a*) Describe all statistical methods, including those used to control for confounding	11-13
		(*b*) Describe any methods used to examine subgroups and interactions	11-13
		(*c*) Explain how missing data were addressed	13
		(*d*) If applicable, explain how loss to follow-up was addressed	13
		(*e*) Describe any sensitivity analyses	n/a
**Results**			
Participants	13*	(a) Report numbers of individuals at each stage of study—eg numbers potentially eligible, examined for eligibility, confirmed eligible, included in the study, completing follow-up, and analysed	n/a
		(b) Give reasons for non-participation at each stage	n/a
		(c) Consider use of a flow diagram	n/a
Descriptive data	14*	(a) Give characteristics of study participants (eg demographic, clinical, social) and information on exposures and potential confounders	n/a
		(b) Indicate number of participants with missing data for each variable of interest	n/a
		(c) Summarise follow-up time (eg, average and total amount)	n/a
Outcome data	15*	Report numbers of outcome events or summary measures over time	n/a

For peer review only - http://bmjopen.bmj.com/site/about/guidelines.xhtml

Main results	16	(*a*) Give unadjusted estimates and, if applicable, confounder-adjusted estimates and their precision (eg, 95% confidence interval). Make clear which confounders were adjusted for and why they were included	n/a
		(*b*) Report category boundaries when continuous variables were categorized	n/a
		(*c*) If relevant, consider translating estimates of relative risk into absolute risk for a meaningful time period	n/a
Other analyses	17	Report other analyses done—eg analyses of subgroups and interactions, and sensitivity analyses	n/a
**Discussion**			
Key results	18	Summarise key results with reference to study objectives	n/a
Limitations	19	Discuss limitations of the study, taking into account sources of potential bias or imprecision. Discuss both direction and magnitude of any potential bias	3, 14
Interpretation	20	Give a cautious overall interpretation of results considering objectives, limitations, multiplicity of analyses, results from similar studies, and other relevant evidence	16
Generalisability	21	Discuss the generalisability (external validity) of the study results	6, 16
**Other information**			
Funding	22	Give the source of funding and the role of the funders for the present study and, if applicable, for the original study on which the present article is based	19

*Give information separately for exposed and unexposed groups.

Note: An Explanation and Elaboration article discusses each checklist item and gives methodological background and published examples of transparent reporting. The STROBE checklist is best used in conjunction with this article (freely available on the Web sites of PLoS Medicine at http://www.plosmedicine.org/, Annals of Internal Medicine at http://www.annals.org/, and Epidemiology at http://www.epidem.com/). Information on the STROBE Initiative is available at http://www.strobe-statement.org.

For peer review only
